# Context-aware simulation enables systematic optimization of long-read mapping parameters

**DOI:** 10.1093/gigascience/giag079

**Published:** 2026-07-08

**Authors:** Jiang Hu, Dongming Fang, Xin Jin, Chentao Yang

**Affiliations:** BGI Research, Wuhan 430074, China; State Key Laboratory of Genome and Multi-omics Technologies, BGI Research, Shenzhen 518083, China; Center for Evolutionary Biology, School of Life Sciences, Fudan University, Shanghai 200438, China; State Key Laboratory of Genome and Multi-omics Technologies, BGI Research, Shenzhen 518083, China; State Key Laboratory of Genome and Multi-omics Technologies, BGI Research, Shenzhen 518083, China; State Key Laboratory of Genome and Multi-omics Technologies, BGI Research, Shenzhen 518083, China; Guangdong Provincial Key Laboratory of Genome Read and Write, BGI Research, Shenzhen 518083, China

**Keywords:** long-read sequencing, long-read simulator, context-aware simulation, parameter tuning, Bayesian optimization

## Abstract

Long-read mapping performance is critical for downstream genomic analyses but remains sensitive to parameter selection. We present CycSim, a context-aware long-read simulator that learns sequence-context-dependent error profiles from empirical data and generates realistic simulated reads. CycSim more faithfully recapitulated real long-read characteristics than existing simulators, providing a high-fidelity simulation framework with known ground truth. Using this framework, we identified a Cyclone-specific parameter set that achieved 2.78-fold faster mapping than an ONT-oriented baseline while maintaining comparable variant-calling performance. For SV-oriented optimization, CycSim-guided refinement improved mapping efficiency by 8.14–34.16% across ONT, HiFi, and Cyclone HG002 datasets; increased SV F1 scores by 0.57–1.75 percentage points; and showed consistent improvements across independent benchmark datasets and different SV callers. Together, these results demonstrate the utility of CycSim for platform- and analysis-goal-specific algorithm development, benchmarking, and parameter optimization.

## Introduction

The accuracy of long-read sequence mapping is fundamental to genomic analysis but is sensitive to parameterization [1–3]. Default parameters offer convenience but are often suboptimal, failing to generalize across sequencing platforms such as PacBio high-fidelity (HiFi) [4], Oxford Nanopore Technologies (ONT) [5], and Cyclone [6], or across analytical objectives such as structural variant (SV) detection [7]. Systematic optimization is impeded by the empirical nature of default parameters, the scarcity of datasets with known ground-truth alignments, and the lack of a comprehensive framework for identifying optimal settings for specific data types or analyses. Simulation-based evaluation offers a practical solution, yet existing long-read simulators largely introduce errors at random, failing to capture sequence-context-dependent biases and global error rate heterogeneity characteristic of real data [8–10]. As a result, current simulators provide limited realism for benchmarking and parameter tuning.

To address this, we developed CycSim, a context-aware long-read simulator that generates reads based on K-mer contexts and error distributions learned from empirical datasets. We integrated CycSim with a Bayesian optimization framework to systematically identify optimal mapping parameters. Benchmarking confirms that CycSim reconstructs error profiles with greater realism than state-of-the-art alternatives. Furthermore, our optimization framework identified superior parameter sets for the Cyclone platform and confirmed the robustness of default settings for HiFi and ONT data. Notably, SV-specific tuning improves both alignment speeds and SV detection accuracy (F1 scores) across all three platforms.

## Methods

### Model training

Reads <10 kb were removed using fxTools (v0.3.1) [18]. The remaining reads from ONT, HiFi, and Cyclone platforms were aligned to the diploid HG002 reference genome using minimap2 (v2.29) with platform-specific presets: ONT (-x lr:hq), HiFi (-x map-hifi), and Cyclone (-k16 -w13 -A2 -B4 -O4,41 -E2,1 -s180 -U70,1000000). Eight chromosomes (1, 2, 17, and 18 from both haplotypes) and their corresponding reads were extracted for platform-specific model training using different simulators.

For CycSim, ONT and Cyclone reads models were trained using cycsim train -r nanopore -t 30 reads.bam Chr1_2_17_18.fa, while HiFi reads models used cycsim train -r hifi -t 30 reads.bam Chr1_2_17_18.fa. NanoSim (v3.2.3) models were trained with read_analysis.py genome --fastq reads.fastq.gz -rg Chr1_2_17_18.fa -t 30 -c. For Badread (v0.4.1), error and quality score models were generated using badread error_model --reference Chr1_2_17_18.fa --reads reads.fastq.gz --alignment map.paf and badread qscore_model --reference Chr1_2_17_18.fa --reads reads.fastq.gz --alignment map.paf. As PBSIM3 does not provide a training module, the pretrained models supplied by the authors for ONT and HiFi data were used directly.

### Data simulation and evaluation

Simulated reads were generated at 20× depth for the same eight HG002 chromosomes using each simulator with its corresponding trained model. CycSim reads were produced using cycsim sim -t 30 -d 20 -c model.cy Chr1_2_17_18.fa. NanoSim reads were simulated with simulator.py genome -rg Chr1_2_17_18.fa -c training -x 20 -t 30 --fastq. For Badread, Cyclone reads were generated using badread simulate --reference Chr1_2_17_18.fa --quantity 20× --error_model badread_errors --qscore_model badread_qscore --length 20000,15000 --identity 97,100,2.5. ONT simulations used an adjusted identity range (20, 3), and HiFi simulations used platform-specific length (15374, 13000) and identity (30, 3) settings. For PBSIM3, ONT reads were produced using pbsim --strategy wgs --method errhmm --errhmm ERRHMM-ONT-HQ.model --depth 20 --genome Chr1_2_17_18.fa --length-mean 20000 --accuracy-mean 0.994 --accuracy-min 0.95 --length-min 5000. HiFi reads were generated with pbsim --strategy wgs --method errhmm --errhmm ERRHMM-SEQUEL.model --depth 20 --genome Chr1_2_17_18.fa --length-mean 15374 --pass-num 15, followed by CCS processing (ccs, default parameters) to produce final HiFi reads. All simulated reads were then aligned to the reference genome using minimap2, and we quantified read length, alignment identity, and error biases for each simulator.

### Alignment parameter optimization

CycSim was used to simulate reads with default settings and platform-specific trained models. Simulated ONT, HiFi, and Cyclone datasets were aligned to the reference genome using minimap2. SVs were called using Sniffles2 (v2.6.3) with the --tandem-repeats option, with additional validation using cuteSV (v2.1.3), and benchmarked against the HG002 GIAB v1.1 truth set [12] using truvari [19] with --passonly -r 1000 --refine. SNP calling was first performed with Longshot, and evaluated using hap.py with the --engine vcfeval [20]. SNPs and Indels were also called with Clair3 (v2.0.0) using default parameters. For Cyclone data, the r941_prom_hac_g360+g422 model was used because no publicly available Cyclone-specific Clair3 model was available.

To provide an additional independent validation dataset while retaining real-read characteristics as much as possible, we constructed a CHM13-based synthetic SV benchmark. HG002-derived SV alleles and sequences were incorporated into the CHM13 reference to generate a variant-integrated reference genome. Real CHM13 HiFi reads, together with ONT Q20 and Cyclone reads simulated from CHM13 by CycSim using default parameters, were aligned to the variant-integrated reference. Because the reads were derived from the original CHM13 genome, reciprocal variants between CHM13 and the variant-integrated reference were used as the synthetic truth set. SVs were called with Sniffles2 and cuteSV and benchmarked using Truvari.

For simulation-based parameter search, each minimap2 configuration was evaluated against the known truth alignment of simulated reads. Only primary alignments were retained. Two metrics were calculated: interval accuracy, defined as the fraction of reads whose predicted reference interval matched the truth interval within 50 bp, and CIGAR-operation accuracy, defined as the fraction of comparable read positions with identical CIGAR-derived operation labels between the predicted and truth alignments. The general-purpose mapping score was calculated as the average of interval accuracy and CIGAR-operation accuracy, and Optuna minimized one minus this score. Mapping runtime was recorded for each configuration and used during empirical screening. The minimap2 search space included -k = 15–19, -w = 10–19, -A = 1–2, -B = 3–9, -O = 4–14,15–49, -E = 2–3,1, -s ∈ {30, 40, 80, 100, 150, 180, 200, 240}, and -U = 10–80,{500, 5000, 50000, 500000, 1000000}. After optimization, the top 40 non-redundant configurations were re-evaluated on 30× real HG002 data from chromosomes 1, 2, 17, and 18 using downstream SNP, Indel, and SV benchmarks. For SV-oriented optimization, the same minimap2 search space was used, but each candidate configuration was evaluated directly using downstream SV-calling performance. For each configuration, SVs were called with Sniffles2 and benchmarked with Truvari, and Optuna minimized one minus the SV F1 score. cuteSV was used for independent caller validation.

The optimal minimap2 parameters identified for SV calling were selected based on improved F1 score and reduced mapping time. For ONT data, the optimized parameters were -k 21 -w 21 -A 1 -B 9 -O 13,44 -E 3,1 -s 30 -U 20,50000. For HiFi data, the optimized SV-calling parameters were -k 23 -w 22 -A 1 -B 9 -O 13,41 -E 3,1 -s 180 -U 10,5000. For Cyclone data, the optimal parameters for general alignment were -k 16 -w 13 -A 2 -B 4 -O 4,41 -E 2,1 -s 180 -U 70,1000000, which substantially reduced mapping time while maintaining comparable alignment accuracy. In contrast, the Cyclone parameters optimized specifically for SV calling were -k 17 -w 13 -A 1 -B 9 -O 13,44 -E 3,1 -s 30 -U 70,500, which achieved a higher SV F1 score together with faster mapping.

## Results

### Context-aware long-read simulation

CycSim operates through a dual-stage framework comprising model training and read simulation (Supplementary Fig. S1). During the training stage, the algorithm characterizes read structure, including strand orientation, chimerism, aligned/unaligned lengths, and alignment identity by interrogating high-confidence alignments from input BAM files. To mitigate artifacts arising from alignment heuristics, aligned regions are re-aligned using edlib [11] and low-confidence termini are trimmed. Crucially, we model error characteristics at two complementary levels: (1) K-mer-based error modeling, which captures the empirical frequency of context-dependent substitution, insertion, and deletion via a sliding window approach; and (2) error transition modeling, which estimates the transition probabilities between consecutive error states to reflect the local continuity of error types.

In the simulation stage, these models define the genomic origin, structural composition, and expected error rate of synthetic reads. The aligned core is generated through a base-wise sliding process, in which errors are sampled according to the K-mer-specific models and the empirical error transition matrix. Subsequently, Phred-scaled quality scores are assigned, and unaligned regions are appended to form complete reads. When the simulated error rate deviates markedly from the expected value, resampling is performed, and chimeric reads are constructed by concatenating independent simulated fragments.

**Figure 1:**
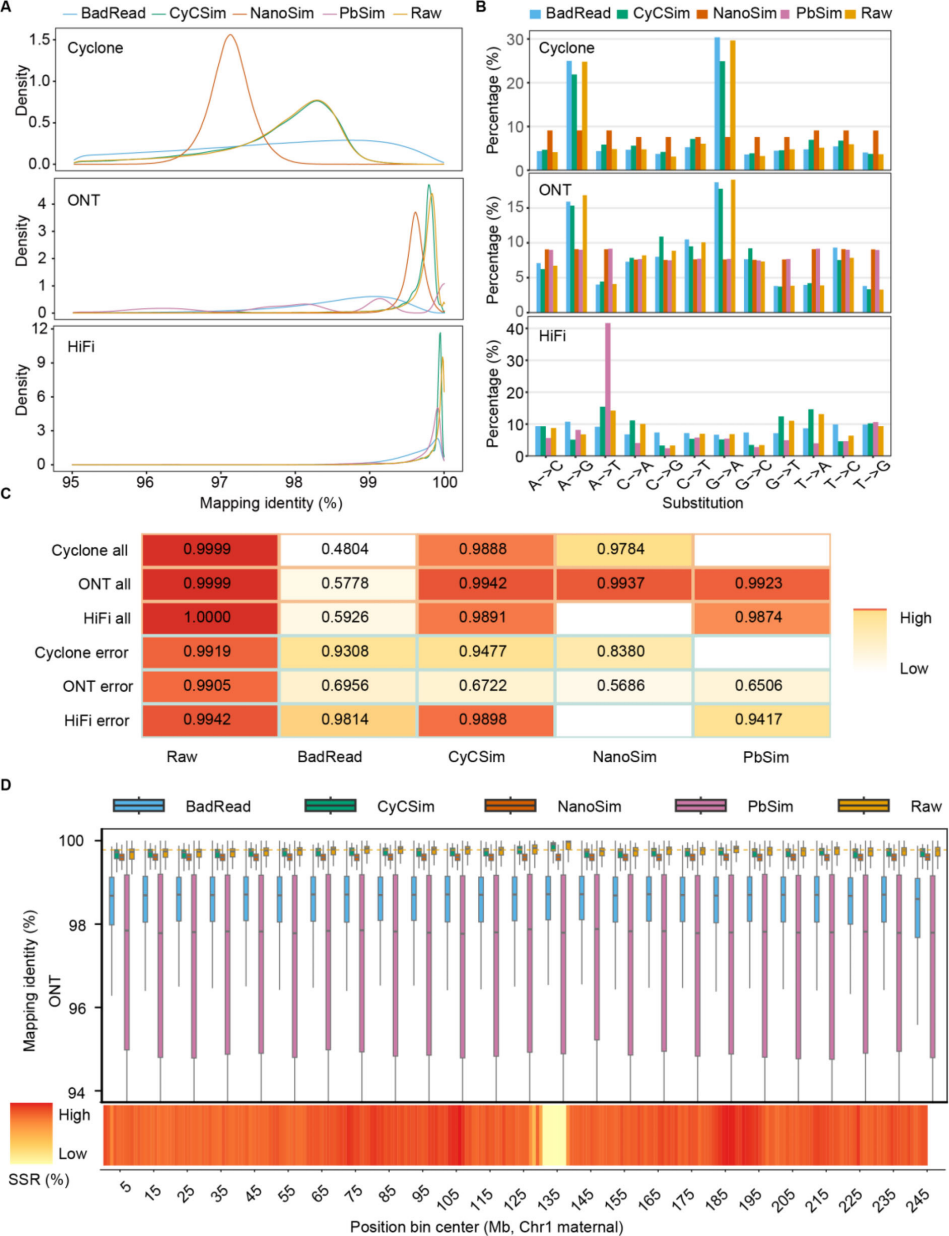
Multidimensional evaluation of long-read simulators. (A) Distribution of alignment identity for simulated reads compared with real reads (Raw). (B) Statistics of substitution error bias of long-read simulators. (C) Cosine similarity of K-mer count between simulated and real reads. All denotes similarity computed over all K-mers; Error denotes K-mers present in the real reads but absent from the reference genome, representing sequencing-induced errors. For Raw, real reads were randomly split into two parts to estimate an empirical upper bound of similarity. Blank entries indicate missing values. (D) Positional alignment identity distribution along Chr1 maternal for simulated ONT reads and real reads. Horizontal yellow lines mark the median identity of real ONT reads. The lower heatmap shows the short tandem repeats (STRs) density (1–6 bp motifs, ≥3 repeat units) along Chr1 maternal. Note that genomic regions experiencing a localized dip or fragmentation in actual STR density naturally correspond to elevated empirical and simulated mapping identities, demonstrating CycSim’s capacity to capture fine-grained positional heterogeneity faithfully.

**Figure 2:**
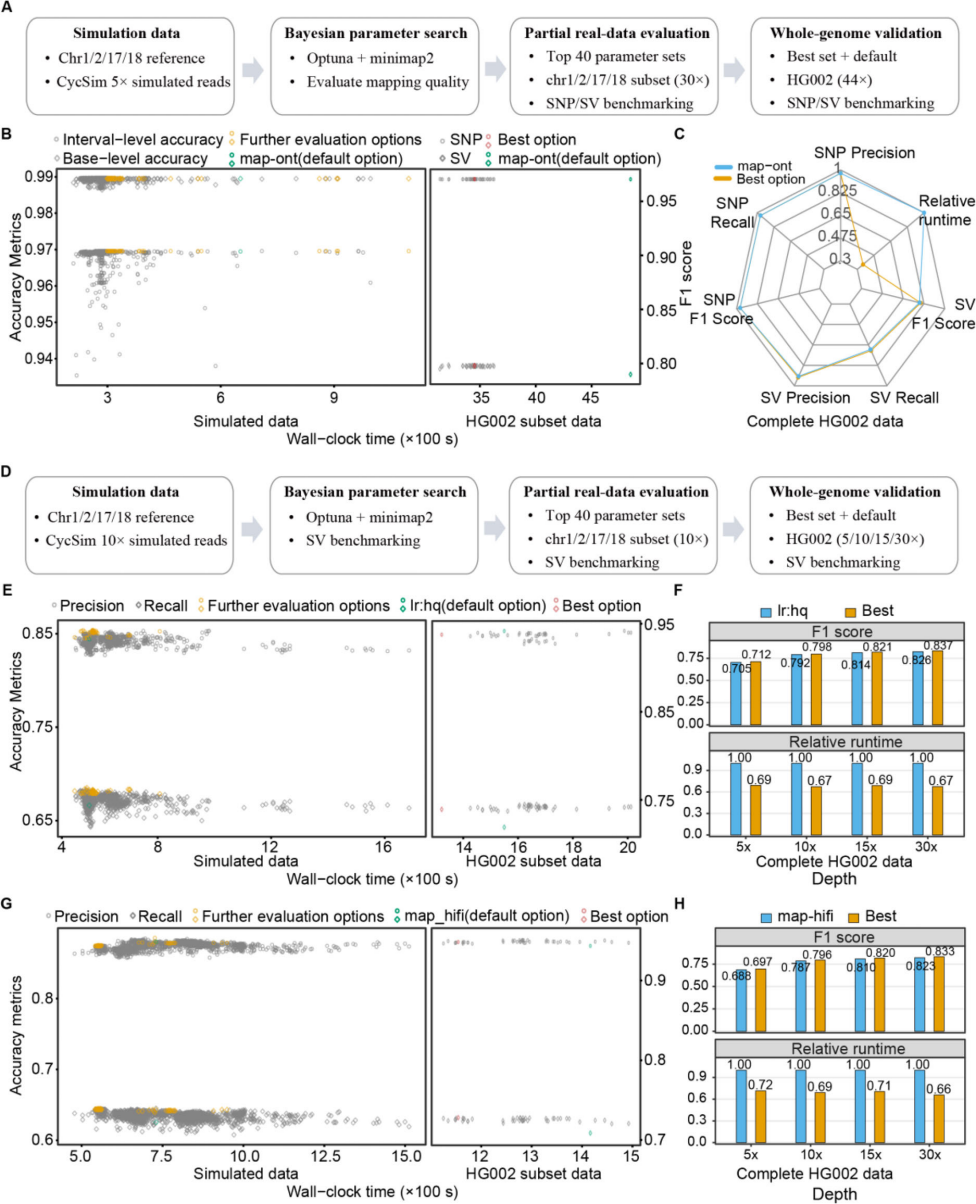
Bayesian optimization and evaluation of mapping parameters. (A) Optimization workflow for general-purpose mapping parameters. (B) Performance of Cyclone mapping parameters on simulated and partial real reads. Interval-level accuracy denotes the proportion of aligned intervals within 50 bp of the true interval, and Base-level accuracy denotes the proportion of correctly aligned bases. SNP and SV accuracy were assessed as described in the “Methods” section. (C) Performance of optimized versus default parameters on 44× real Cyclone reads. (D) Optimization workflow for SV detection-oriented mapping parameters using sniffles. (E) Performance of ONT SV detection-oriented mapping parameters on simulated and partial real reads. (F) Performance of optimized versus default ONT mapping parameters for SV detection at different coverage depths. (G) Performance of HiFi SV detection-oriented mapping parameters on simulated and partial real reads. (H) Performance of optimized versus default HiFi mapping parameters for SV detection at different coverage depths. For Panels B, E, and G, parameters evaluated in the right panel were selected from the left panel (labeled as Further evaluation options), and Wall-clock time includes both minimap2 and samtools sort. Relative runtime for the bar charts (Panels F and H) denotes the proportion of runtime relative to the longest run, considering minimap2 only; for the radar chart (Panel C), relative runtime is mapped as an independent axis. In all cases, lower runtime values indicate faster mapping.

To validate the framework, we benchmarked CycSim against BadRead [10], NanoSim [8], and PbSim3 [9] using the diploid HG002 genome [12] (chromosomes 1, 2, 17, and 18) across ONT, HiFi, and Cyclone platforms. CycSim consistently demonstrated superior fidelity in reproducing both read length and global error rate distributions (Fig. 1A and  Supplementary Fig. S2). Our assessment focused on three key metrics: (1) Base substitution profiles: CycSim and BadRead were the only tools to accurately capture empirically observed substitution biases (Fig. 1B); (2) K-mer distribution: CycSim faithfully reproduced the global K-mer frequency landscape. Specifically, it achieved the highest concordance for erroneous K-mers in Cyclone and HiFi data, while performing competitively on ONT (Fig. 1C). For the Raw comparison, real reads were randomly split into two subsets and compared against each other. Because both subsets originated from the same empirical sequencing dataset, their similarity represents an empirical upper bound for the expected concordance between simulated and real reads under finite sampling. (3) Error rates in simple repeats: Notably, real Cyclone and ONT data exhibit substantial error rate heterogeneity in low-complexity regions. While other simulators produced artifactually uniform positional identity profiles, CycSim more accurately recapitulated the regional heterogeneity observed in real reads, including higher alignment identity in regions with lower STR/SSR density (Fig. 1D and Supplementary Fig. S3). Error decomposition and region-stratified substitution analysis further showed that the low-complexity-associated error increase in real Cyclone and ONT data was mainly driven by elevated deletion rates and that CycSim recapitulated these profiles across sequence-complexity classes (Supplementary Figs S4–S6). In addition, three independent CycSim simulations using the same trained model and settings produced nearly overlapping mapping identity distributions and highly consistent substitution spectra, supporting the reproducibility of CycSim-generated read profiles (Supplementary Fig. S7). Finally, applying the HG002-trained CycSim model to an independent HG005 Cyclone dataset yielded simulated reads with alignment identity distributions and substitution profiles similar to real HG005 reads, supporting model generalization beyond the training sample (Supplementary Fig. S8).

Collectively, these analyses demonstrate that CycSim provides an HiFi, platform-consistent representation of long-read characteristics, capturing both global and localized, context-dependent error biases that are insufficiently modeled by existing simulators. Nevertheless, CycSim is expected to perform best when the training data are generated from the same or a closely related genome, sequencing platform, chemistry, and library preparation protocol as the intended simulation target.

### Bayesian optimization of mapping parameters

HiFi simulation is a prerequisite for simulation-guided optimization because an optimizer may otherwise overfit to simulator-specific artifacts, such as artificially uniform error distributions or missing sequence-context-dependent biases. Leveraging the improved realism of CycSim-generated reads, we established a four-stage Bayesian optimization framework to systematically identify optimal mapping parameters. The workflow proceeds as follows: (1) Simulation-based initialization using CycSim-generated reads with known ground-truth coordinates; (2) Bayesian parameter search via Optuna [13], which explores thousands of minimap2 [2] configurations to maximize a defined objective function; (3) Empirical screening, where top-performing parameter sets are evaluated on real data subsets; (4) Whole-genome validation to ensure robustness across full-scale datasets.

We first applied the framework to optimize general-purpose alignment (Fig. 2A). The optimization utilized 5× CycSim-simulated HG002 data (Chr 1, 2, 17, 18) to maximize a composite accuracy metric integrating both base-level identity and interval-level overlap. From this search, the top 40 configurations were screened using 30× real HG002 data on the same chromosomal subset, assessed using standard SNP, Indel, and SV benchmarks. The optimal configuration was subsequently validated on 44× whole-genome data.

When applied to the emerging Cyclone platform, using minimap2’s ONT-oriented map-ont preset as an initial baseline, our framework identified a novel parameter set that increased mapping speed by 2.78-fold while maintaining comparable small-variant calling accuracy and modestly improving SV detection (Fig. 2B and C, Supplementary Tables S1–S3). Importantly, the parameter search was performed only on HG002 chromosomes 1, 2, 17, and 18, whereas generalization was evaluated on held-out HG002 chromosomes excluding chromosomes 1, 2, 17, and 18, whole-genome HG002 data. For small variants, independent-sample validation was further performed using an HG005 dataset, whereas SV-oriented validation was extended using an independent CHM13-based synthetic SV benchmark. Small-variant accuracy was assessed using the deep-learning-based caller Clair3 [14] for SNP and Indel evaluation, together with the haplotype-aware statistical caller Longshot [15] for independent SNP validation. Across these validation settings, the optimized parameters maintained comparable SNP/Indel accuracy while substantially reducing mapping runtime and improving SV F1 scores with both Sniffles2 [16] and cuteSV [17], two widely used long-read SV callers (Supplementary Tables S1–S3). Conversely, for HiFi and ONT datasets, the framework identified configurations with performance close to the established minimap2 default presets. Because these presets have already been extensively optimized for mature long-read platforms, only marginal accuracy gains (<0.1% in simulation) with slightly reduced runtime were observed (Supplementary Fig. S9). These results indicate that the framework can recover parameter settings comparable to developer-optimized defaults when existing presets are already well matched to the data, while still enabling parameter refinement for datasets with distinct read characteristics.

We next adapted the framework specifically for SV detection, for which breakpoint placement and interval-level alignment consistency are critical (Fig. 2D). Using the same training and validation design described above, we modified the optimization objective to maximize the SV F1 score and minimize mapping runtime and evaluated the resulting parameter sets with Sniffles2 and cuteSV.

Across ONT, HiFi, and Cyclone datasets, SV-oriented optimization yielded more efficient mapping configurations while preserving or improving SV calling accuracy. On whole-genome HG002 datasets at varying depths, and across both Sniffles2 and cuteSV, the optimized parameters achieved 30.84–32.65% faster mapping and 0.57–1.12 percentage-point higher SV F1 scores for ONT data, 28.34–34.16% faster mapping and 0.77–1.03 percentage-point higher SV F1 scores for HiFi data, and 8.14–9.83% faster mapping and 0.59–1.75 percentage-point higher SV F1 scores for Cyclone data relative to the corresponding default or baseline settings (Fig. 2E–H and Supplementary Fig. S10 and  Tables S4–S9).

Validation on held-out HG002 chromosomes excluding chromosomes 1, 2, 17, and 18, together with validation on an independent CHM13-based synthetic SV benchmark, showed the same overall trend across platforms, coverage depths, and both SV callers (Supplementary Tables S4–S9). These results indicate that the SV-oriented parameters were not specific to the HG002 training chromosomes, the HG002 sample, or a single downstream SV-calling method. Together, these findings support the utility of CycSim-guided optimization for deriving task-specific mapping parameters that improve SV analysis while reducing computational cost.

## Discussion

Several practical considerations and limitations should be noted. First, when simulated depth substantially exceeds training depth, learned error contexts may be over-represented because k-mer-specific error models are estimated from a finite training set. CycSim provides a user-defined parameter to introduce random errors according to the global error-rate distribution, increasing error-pattern diversity at high simulated coverage. For k-mer contexts absent from the training data, CycSim automatically falls back to the global average error model. These strategies preserve the overall error level, although some fine-scale context-specific biases may still be missed.

Second, the optimized parameters are task-specific. SV-oriented parameters improved SV calling and reduced mapping time, but they did not consistently improve SNP and indel calling across all settings (Supplementary Table S10). This trade-off underscores the alignment heuristics where parameters relaxed to capture large structural disruptions can occasionally sacrifice single-base resolution. To facilitate practical applications, we propose a best practice guideline for parameter selection: for large-scale sequencing cohorts where SV screening is the primary objective, the SV-optimized preset provides optimal throughput and sensitivity; conversely, for comprehensive pipelines targeting a full spectrum of genomic variations (including SNPs and Indels), the CycSim-derived general-purpose preset should be prioritized to ensure well-balanced accuracy across all variant scales.

Third, optimized parameters should be selected according to the application scenario. Minimap2 default presets remain strong general-purpose choices, especially for mature ONT and HiFi datasets. However, they may be less optimal for emerging platforms, datasets with distinct read characteristics, or analysis-specific goals such as SV detection. In these settings, parameter refinement can improve the trade-off between downstream performance and computational efficiency. Although the SV F1-score gains were modest, the runtime reductions were substantial, making the SV-oriented parameters valuable for population-scale long-read projects where per-sample speedups accumulate across large cohorts.

Fourth, the full optimization process is computationally intensive and is therefore most suitable for large-scale or repeatedly used workflows, where the upfront search cost can be amortized across many samples (Supplementary Table S11), or for software developers aiming to establish optimized default parameters for specific platforms and analysis tasks. Finally, the optimization framework is modular and can in principle incorporate reads generated by other simulators. Because simulation-guided optimization depends on how well simulated reads reflect real read characteristics, parameters selected from any simulator should still undergo empirical screening, independent validation, and, where necessary, additional diagnostic evaluation to avoid simulator-specific biases. More broadly, this framework provides a reproducible strategy for analysis-goal-driven parameter refinement by combining simulation-guided candidate generation with real-data validation.

## Conclusions

We have developed CycSim, a context-aware simulator that faithfully reproduces the complex error characteristics of long-read sequencing data. We paired this with an analysis-goal-driven Bayesian optimization framework that enables systematic refinement of mapping parameters. Together, these tools provide a robust foundation for improving the accuracy and efficiency of long-read analyses and optimizing bioinformatics workflows for specific platforms and analytical goals.

## Availability of source code and requirements

- Project name: CycSim
- Project home page: https://github.com/BioEarthDigital/CycSim
- Operating system(s): Linux
- Programming language: Rust
- License: MIT
- RRID:SCR_028425
- Biotools:cycsim

## Additional files

**Supplementary Fig. S1:** CycSim pipeline. The CycSim pipeline operates in two stages: model training and read simulation. The training stage processes high-confidence BAM alignments to characterize read structure and models error characteristics at two complementary levels: K-mer-based modeling and Error transition modeling. In the simulation stage, these derived models define the read’s structure and error profile, and the aligned read core is generated via a base-wise sliding process that samples errors using both models. A detailed description is available in the “Context-aware long-read simulation” section.

**Supplementary Fig. S2:** Distribution of reads length of simulated reads compared with real reads.

**Supplementary Fig. S3:** Positional alignment identity distribution along Chr1 maternal for simulated Cyclone and HiFi reads. Horizontal yellow lines mark the median identity of real ONT reads. The lowest heatmap shows the short tandem repeats (STRs) density (1–6 bp motifs, ≥3 repeat units) along Chr1 maternal.

**Supplementary Fig. S4:** Sequence-complexity-stratified error profiles of real and simulated Cyclone reads. (A) Difference in total alignment-derived error rate between low- and high-complexity regions. Positive values indicate increased error rates in low-complexity regions. (B) Decomposition of the low-minus-high error-rate difference into substitutions, insertions, and deletions. (C) Substitution spectra in high- and low-complexity regions, shown as the fraction of each substitution type among all substitutions. The reference genome was divided into non-overlapping 10-kb windows and ranked by the fraction of bases covered by short tandem-repeat-like sequence contexts, defined as 1–6 bp motifs repeated at least three times. Windows in the lowest 5% and highest 5% of repeat-content fraction were defined as high-complexity and low-complexity regions, respectively.

**Supplementary Fig. S5:** Sequence-complexity-stratified error profiles of real and simulated HiFi reads. (A) Difference in total alignment-derived error rate between low- and high-complexity regions. Positive values indicate increased error rates in low-complexity regions. (B) Decomposition of the low-minus-high error-rate difference into substitutions, insertions, and deletions. (C) Substitution spectra in high- and low-complexity regions, shown as the fraction of each substitution type among all substitutions. The reference genome was divided into non-overlapping 10-kb windows and ranked by the fraction of bases covered by short tandem-repeat-like sequence contexts, defined as 1–6 bp motifs repeated at least three times. Windows in the lowest 5% and highest 5% of repeat-content fraction were defined as high-complexity and low-complexity regions, respectively.

**Supplementary Fig. S6:** Sequence-complexity-stratified error profiles of real and simulated ONT reads. (A) Difference in total alignment-derived error rate between low- and high-complexity regions. Positive values indicate increased error rates in low-complexity regions. (B) Decomposition of the low-minus-high error-rate difference into substitutions, insertions, and deletions. (C) Substitution spectra in high- and low-complexity regions, shown as the fraction of each substitution type among all substitutions. The reference genome was divided into non-overlapping 10-kb windows and ranked by the fraction of bases covered by short tandem-repeat-like sequence contexts, defined as 1–6 bp motifs repeated at least three times. Windows in the lowest 5% and highest 5% of repeat-content fraction were defined as high-complexity and low-complexity regions, respectively.

**Supplementary Fig. S7:** Reproducibility of CycSim simulations across independent replicates. (A) Distribution of alignment identity for simulated reads compared with real reads (Raw). (B) Statistics of substitution error bias for simulated reads compared with real reads (Raw). Rep1, Rep2, and Rep3 represent three independent simulation replicates.

**Supplementary Fig. S8:** Generalization of the HG002-trained CycSim model to an independent HG005 dataset using Cyclone reads. (A) Distribution of alignment identity for CycSim-simulated and real reads. (B) Substitution error profiles of simulated and real reads.

**Supplementary Fig. S9:** Performance of HiFi and ONT mapping parameters on simulated reads. Interval-level accuracy denotes the proportion of aligned intervals within 50 bp of the true interval, and Base-level accuracy denotes the proportion of correctly aligned bases.

**Supplementary Fig. S10:** Bayesian optimization and evaluation of mapping parameters. (A) Performance of Cyclone SV detection-oriented mapping parameters on simulated and partial real reads. Parameters evaluated in the right panel were selected from the left panel (labeled as Further evaluation options), and Wall-clock time includes both minimap2 and samtools sort. The default option represents the optimal parameters we identified for general-purpose mapping. (B) Performance of optimized versus default Cyclone mapping parameters for SV detection at different coverage depths using sniffles. Relative runtime denotes the proportion of runtime relative to the longest run, considering minimap2 only.

**Supplementary Table S1:** Clair3-based SNP and INDEL calling performance for Cyclone data using default and optimized mapping parameters.

**Supplementary Table S2:** Longshot-based SNP calling performance for Cyclone data using default and optimized mapping parameters.

**Supplementary Table S3:** SV calling performance for Cyclone data using default and optimized mapping parameters.

**Supplementary Table S4:** Coverage-dependent SV calling performance for HG002 ONT data using default and optimized minimap2 parameters.

**Supplementary Table S5:** SV calling performance for CHM13-based synthetic ONT data using default and optimized minimap2 parameters.

**Supplementary Table S6:** Coverage-dependent SV calling performance for HG002 HiFi data using default and optimized minimap2 parameters.

**Supplementary Table S7:** SV calling performance for CHM13-based synthetic HiFi data using default and optimized minimap2 parameters.

**Supplementary Table S8:** Coverage-dependent SV calling performance for HG002 Cyclone data using default and optimized minimap2 parameters.

**Supplementary Table S9:** SV calling performance for CHM13-based synthetic Cyclone data using default and optimized minimap2 parameters.

**Supplementary Table S10:** Clair3-based SNP and INDEL calling performance using default and SV-optimized mapping parameters.

**Supplementary Table S11:** Computational resource usage of mapping parameter optimization for Cyclone data.

## Consent for publication

Not applicable.

## Ethics approval and consent to participate

Not applicable.

## Supplementary Material

giag079_Supplemental_File

giag079_Authors_Response_To_Reviewer_Comments_Original_Submission

giag079_Authors_Response_To_Reviewer_Comments_revision_1

giag079_GIGA-D-26-00085_original_submission

giag079_GIGA-D-26-00085_Revision_1

giag079_GIGA-D-26-00085_Revision_2

giag079_Reviewer_1_Report_Original_Submission Reviewer 1 -- 4/2/2026

giag079_Reviewer_1_Report_Revision_1 Reviewer 1 -- 6/17/2026

giag079_Reviewer_2_Report_Original_Submission Reviewer 2 -- 4/15/2026

## Data Availability

The HG002 v1.1 reference genome and HiFi reads were obtained from the HG002 repository [21]. The HG002 ONT Q20 dataset was downloaded from the ONT Open Datasets portal [22]. HG002 Cyclone reads were retrieved from the China National GeneBank (CNGB) under accession CNP0007646, whereas the HG005 Cyclone reads were obtained from the official Cyclone team upon request. The CHM13 reference genome and HiFi reads were downloaded from the CHM13 repository [23]. The HG002 small-variant and structural-variant benchmark sets, together with the HG005 small-variant benchmark set, were downloaded from Genome in a Bottle (GIAB). CycSim and its pretrained models are released under the Massachusetts Institute of Technology (MIT) License and are available on GitHub [24] and Zenodo [25].
