# Supplementary material for: Context-aware simulation enables systematic optimization of long-read mapping parameters
Source: Gigascience. 2026 Jul 8;15:giag079. doi: 10.1093/gigascience/giag079 (PMC13401045; doi:10.1093/gigascience/giag079)
Supplement: giag079_Supplemental_File [file giag079_supplemental_file.docx]

# Supplementary Information

# Context-aware simulation enables systematic optimization of long-read mapping parameters

Jiang Hu^1,2,3^, Dongming Fang^2^, Xin Jin^2^, Chentao Yang^2,4,*^

1. BGI Research, Wuhan 430074, China
2. State Key Laboratory of Genome and Multi-omics Technologies, BGI Research, Shenzhen 518083, China
3. Center for Evolutionary Biology, School of Life Sciences, Fudan University, Shanghai 200438, China
4. Guangdong Provincial Key Laboratory of Genome Read and Write, BGI Research, Shenzhen 518083, China

*Corresponding authors: Chentao Yang: yangchentao@genomics.cn

# Supplementary Figures

**
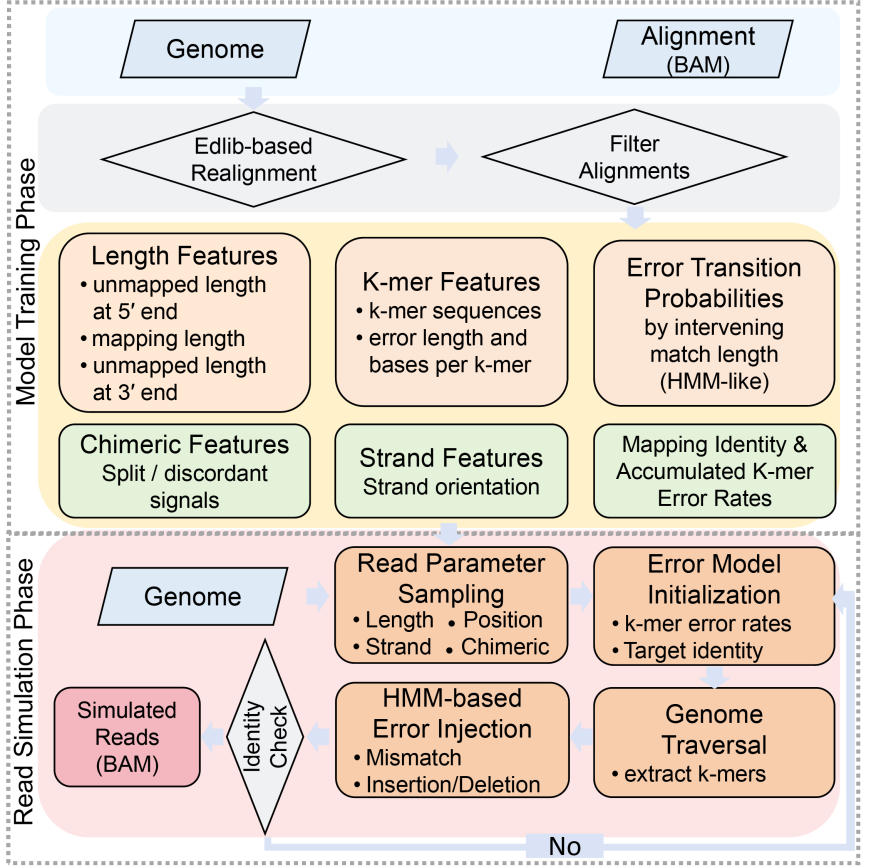
**

**Figure S1** CycSim pipeline. The CycSim pipeline operates in two stages: model training and read simulation. The training stage processes high-confidence BAM alignments to characterize read structure and models error characteristics at two complementary levels: K-mer-based modeling and Error transition modeling. In the simulation stage, these derived models define the read's structure and error profile, and the aligned read core is generated via a base-wise sliding process that samples errors using both models. A detailed description is available in the "Context-aware long-read simulation" section.

**
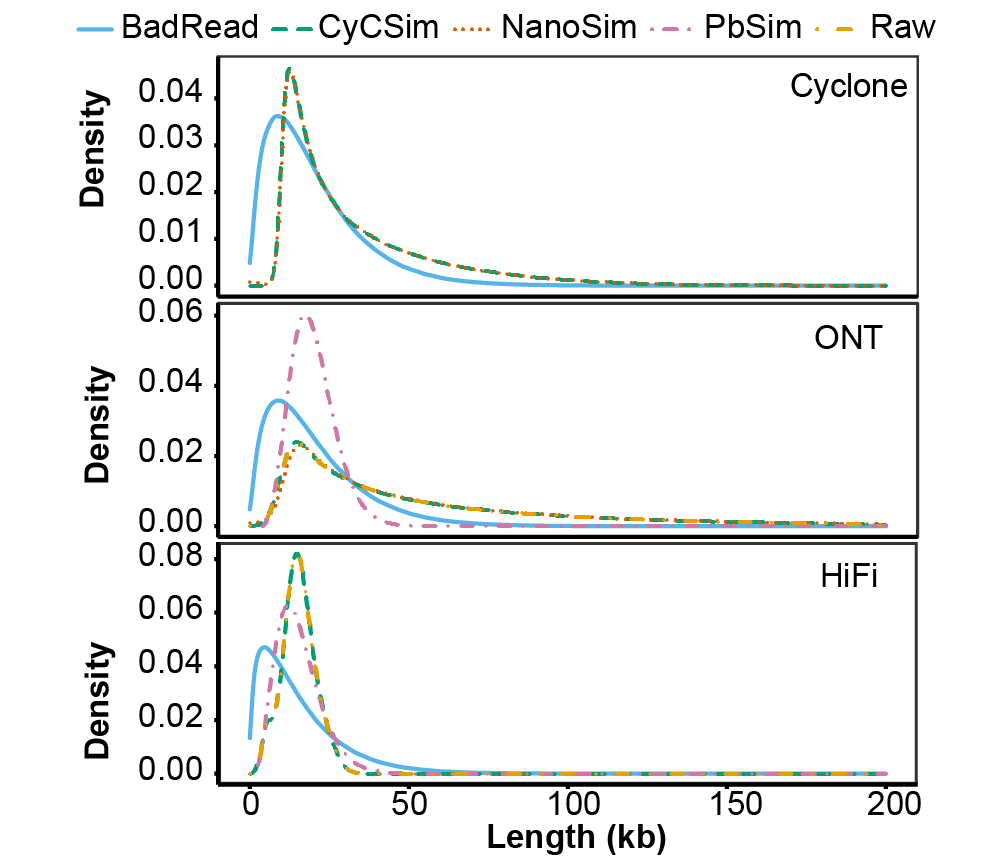
**

**Figure S2** Distribution of reads length of simulated reads compared with real reads.


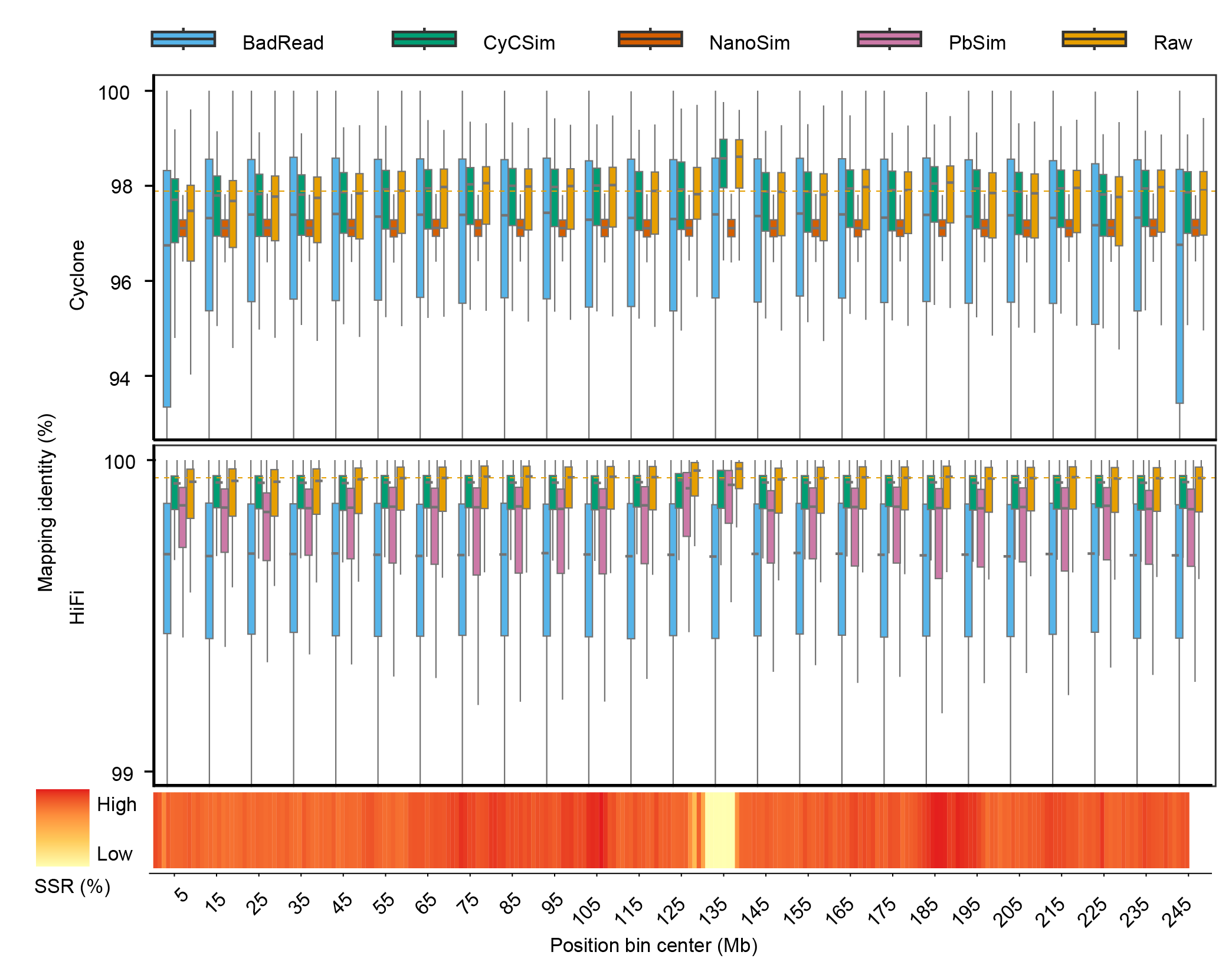


**Figure S3** Positional alignment identity distribution along Chr1 maternal for simulated Cyclone and HiFi reads. Horizontal yellow lines mark the median identity of real ONT reads. The lowest heatmap shows the short tandem repeats (STRs) density (1–6 bp motifs, ≥3 repeat units) along Chr1 maternal.


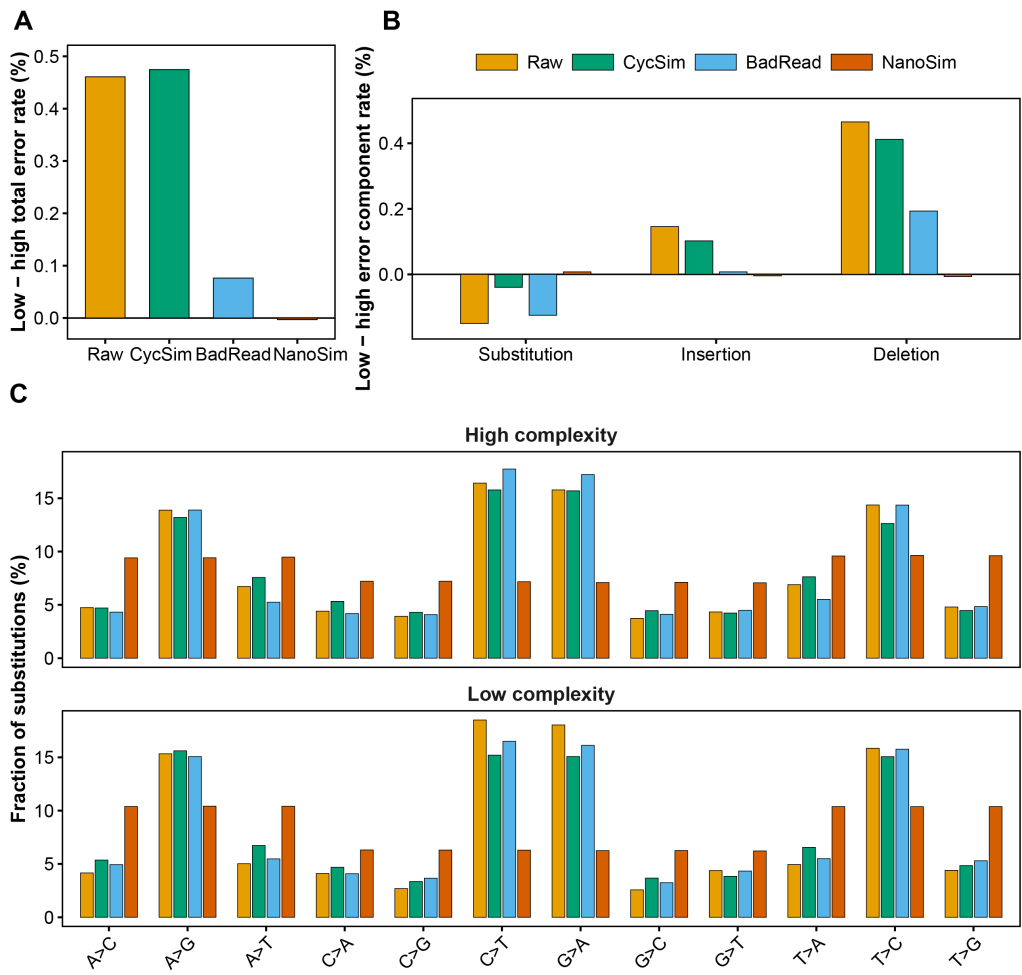


**Figure S4** Sequence-complexity-stratified error profiles of real and simulated Cyclone reads. **A** Difference in total alignment-derived error rate between low- and high-complexity regions. Positive values indicate increased error rates in low-complexity regions. **B** Decomposition of the low-minus-high error-rate difference into substitutions, insertions, and deletions. **C** Substitution spectra in high- and low-complexity regions, shown as the fraction of each substitution type among all substitutions. The reference genome was divided into non-overlapping 10-kb windows and ranked by the fraction of bases covered by short tandem-repeat-like sequence contexts, defined as 1–6 bp motifs repeated at least three times. Windows in the lowest 5% and highest 5% of repeat-content fraction were defined as high-complexity and low-complexity regions, respectively.


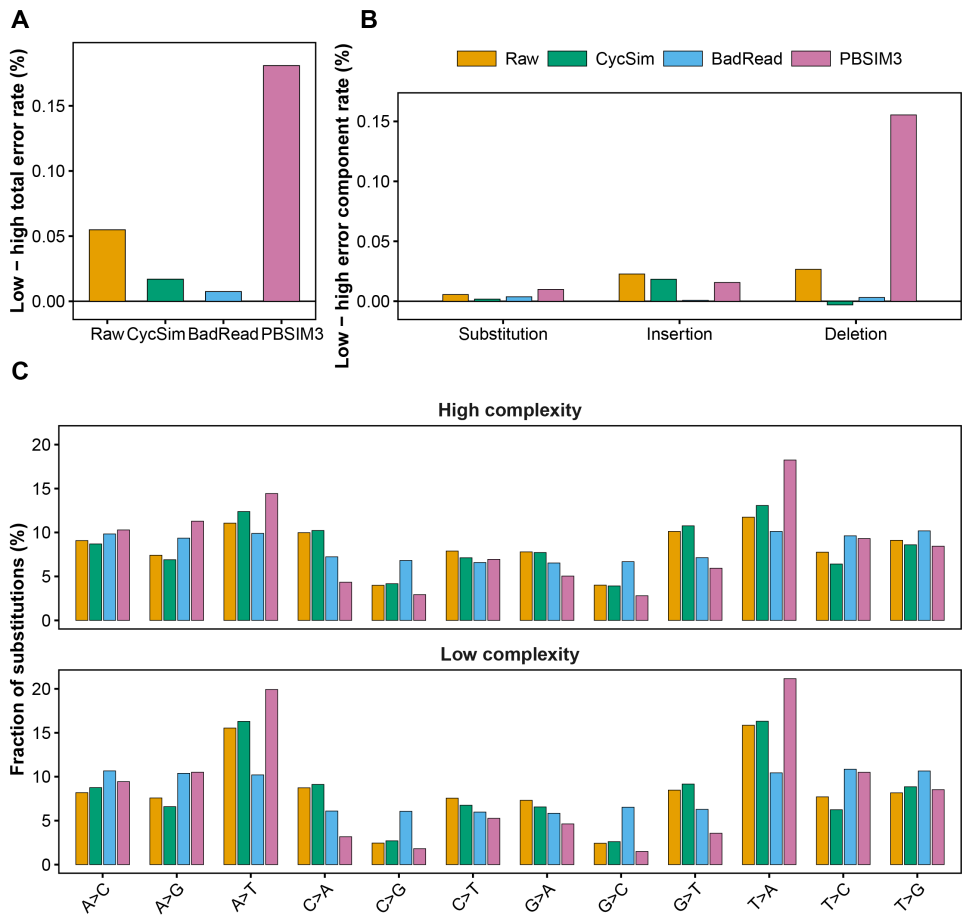


**Figure S5** Sequence-complexity-stratified error profiles of real and simulated HiFi reads. **A** Difference in total alignment-derived error rate between low- and high-complexity regions. Positive values indicate increased error rates in low-complexity regions. **B** Decomposition of the low-minus-high error-rate difference into substitutions, insertions, and deletions. **C** Substitution spectra in high- and low-complexity regions, shown as the fraction of each substitution type among all substitutions. The reference genome was divided into non-overlapping 10-kb windows and ranked by the fraction of bases covered by short tandem-repeat-like sequence contexts, defined as 1–6 bp motifs repeated at least three times. Windows in the lowest 5% and highest 5% of repeat-content fraction were defined as high-complexity and low-complexity regions, respectively.


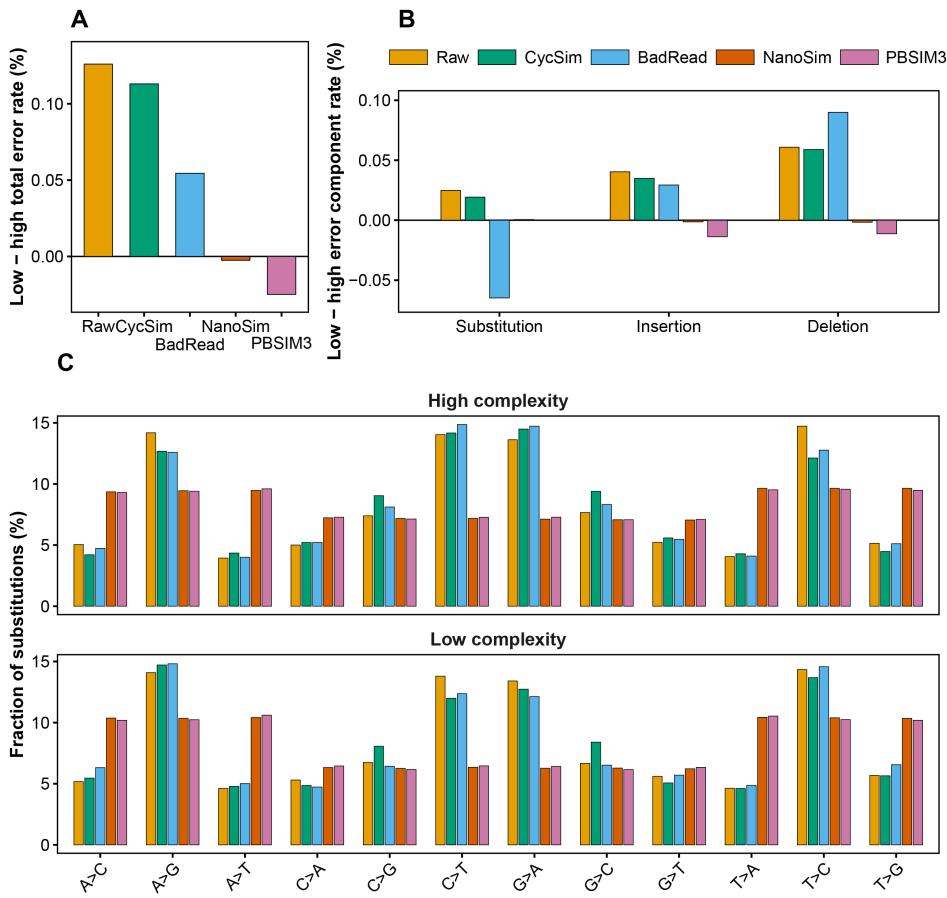


**Figure S6** Sequence-complexity-stratified error profiles of real and simulated ONT reads. **A** Difference in total alignment-derived error rate between low- and high-complexity regions. Positive values indicate increased error rates in low-complexity regions. **B** Decomposition of the low-minus-high error-rate difference into substitutions, insertions, and deletions. **C** Substitution spectra in high- and low-complexity regions, shown as the fraction of each substitution type among all substitutions. The reference genome was divided into non-overlapping 10-kb windows and ranked by the fraction of bases covered by short tandem-repeat-like sequence contexts, defined as 1–6 bp motifs repeated at least three times. Windows in the lowest 5% and highest 5% of repeat-content fraction were defined as high-complexity and low-complexity regions, respectively.


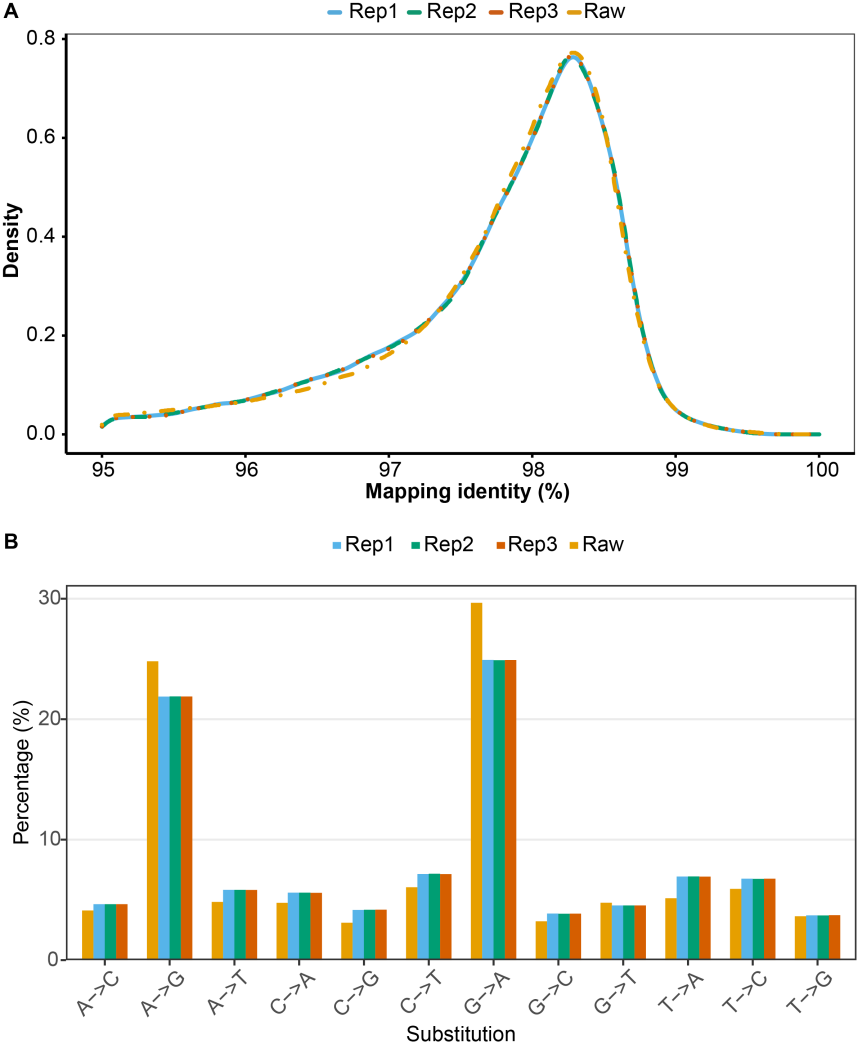


**Figure S7** Reproducibility of CycSim simulations across independent replicates. **A** Distribution of alignment identity for simulated reads compared with real reads (Raw). **B** Statistics of substitution error bias for simulated reads compared with real reads (Raw). Rep1, Rep2, and Rep3 represent three independent simulation replicates.


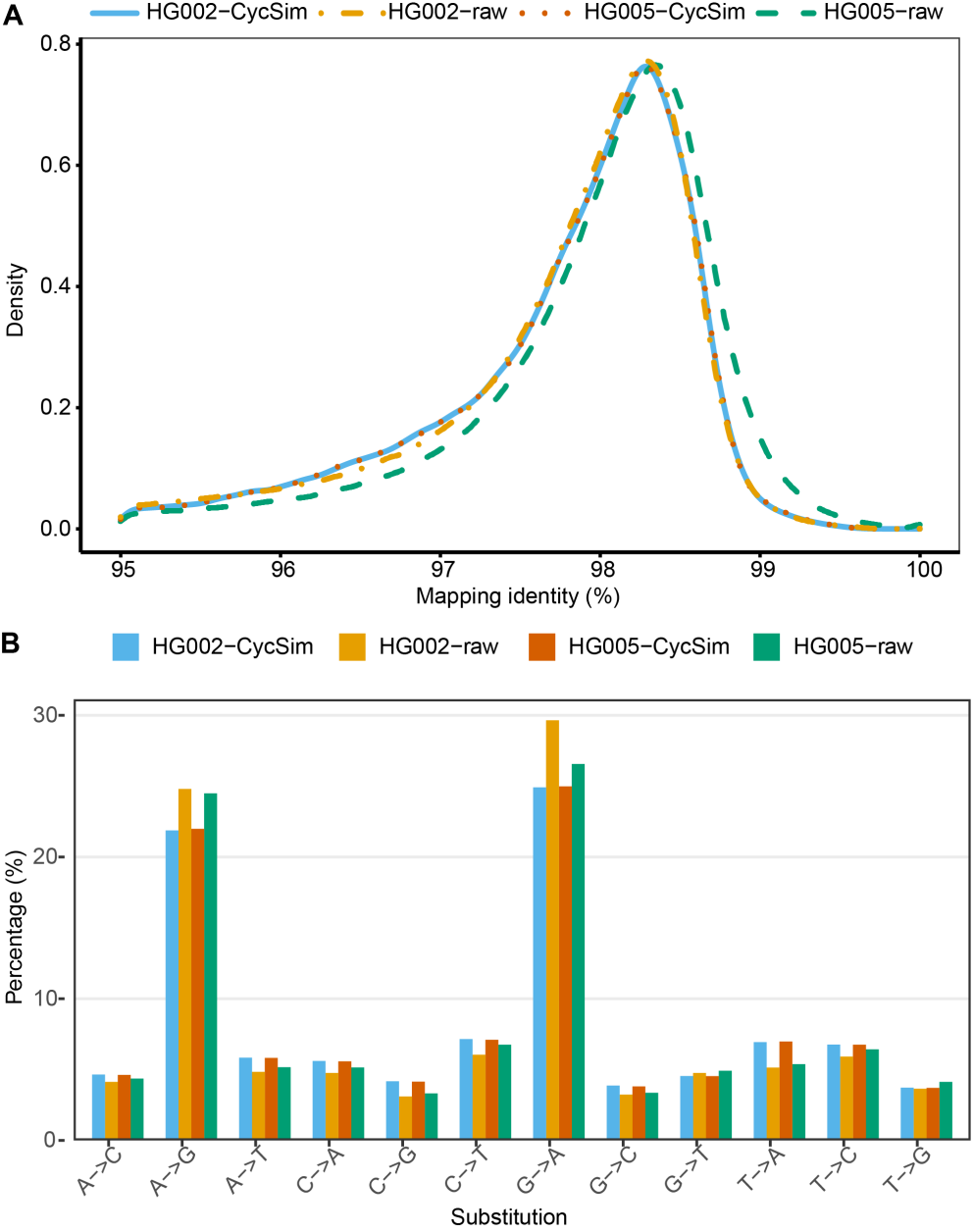


**Figure S8** Generalization of the HG002-trained CycSim model to an independent HG005 dataset using Cyclone reads. **A** Distribution of alignment identity for CycSim-simulated and real reads. **B** Substitution error profiles of simulated and real reads.


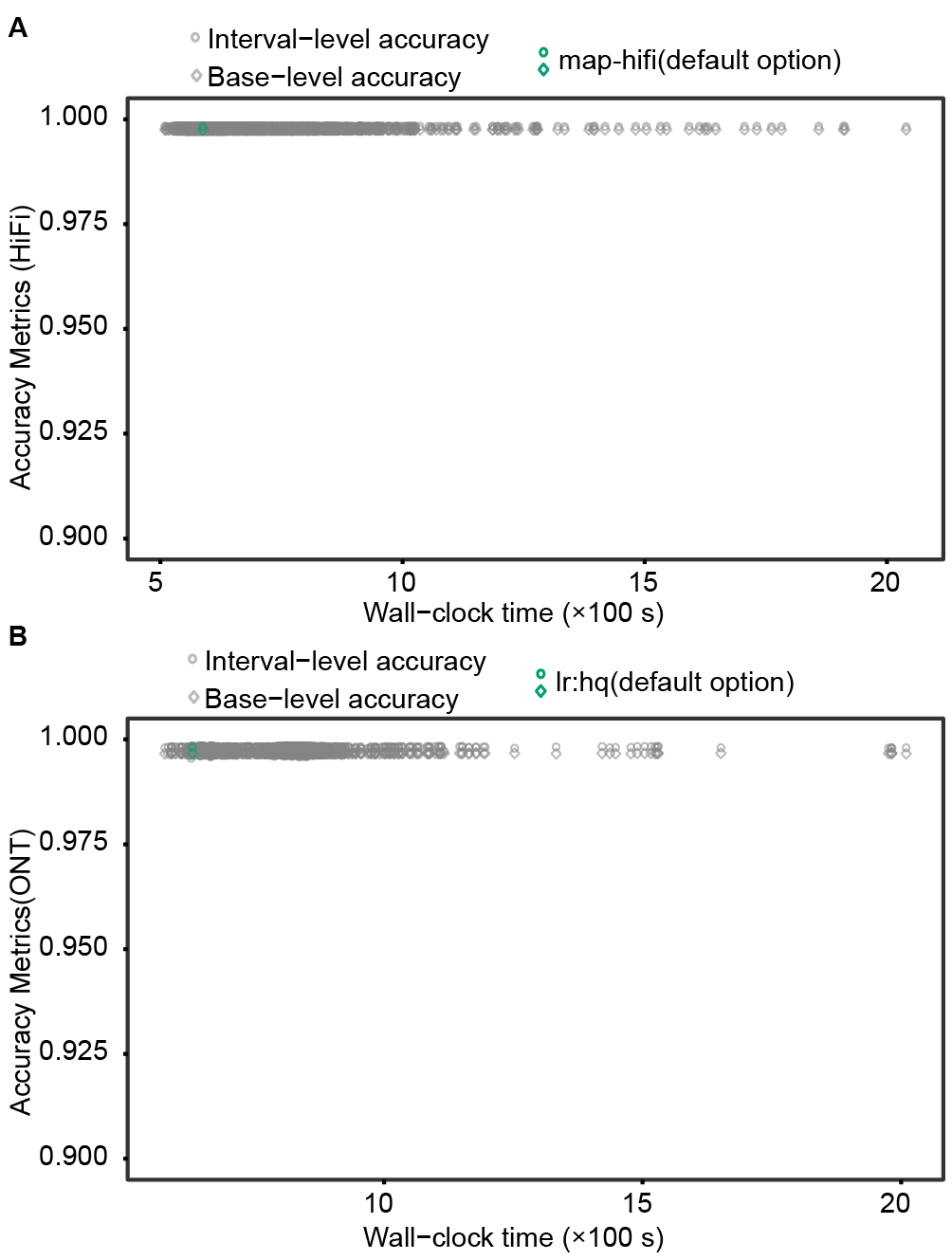


**Figure S9** Performance of HiFi and ONT mapping parameters on simulated reads. Interval-level accuracy denotes the proportion of aligned intervals within 50 bp of the true interval, and Base-level accuracy denotes the proportion of correctly aligned bases.


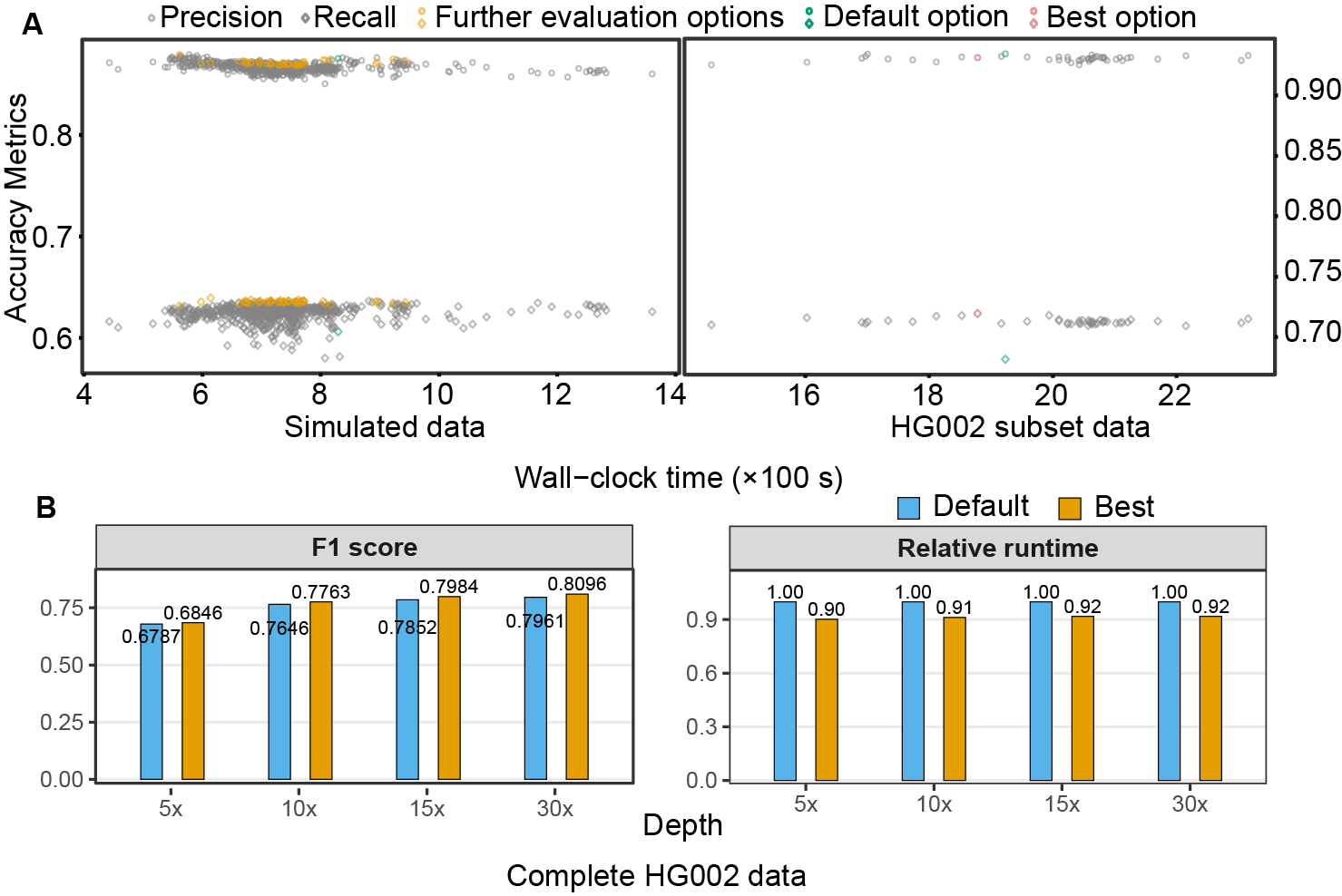


**Figure S10** Bayesian optimization and evaluation of mapping parameters. **A** Performance of Cyclone SV detection-oriented mapping parameters on simulated and partial real reads. Parameters evaluated in the right panel were selected from the left panel (labeled as Further evaluation options), and Wall-clock time includes both minimap2 and samtools sort. The default option represents the optimal parameters we identified for general-purpose mapping. **B** Performance of optimized versus default Cyclone mapping parameters for SV detection at different coverage depths using sniffles. Relative runtime denotes the proportion of runtime relative to the longest run, considering minimap2 only.

# Supplementary Tables

**Table S1** Clair3-based SNP and INDEL calling performance for Cyclone data using default and optimized mapping parameters.

| Sample | Type | Options | Training set (chr1/2/17/18) | | | Held-out set (Non-chr1/2/17/18) | | | Whole genome | | |
| --- | --- | --- | --- | --- | --- | --- | --- | --- | --- | --- | --- |
|  |  |  | Precision | Recall | F1 score | Precision | Recall | F1 score | Precision | Recall | F1 score |
| HG002 | INDEL | map_ont | **0.7660** | **0.4734** | **0.5851** | **0.7674** | **0.4854** | **0.5947** | **0.7671** | **0.4828** | **0.5926** |
|  |  | new_opt | 0.7645 | 0.4726 | 0.5841 | 0.7660 | 0.4847 | 0.5937 | 0.7656 | 0.4820 | 0.5916 |
|  | SNP | map_ont | **0.9928** | 0.9854 | **0.9891** | **0.9906** | **0.9857** | **0.9881** | **0.9911** | 0.9856 | **0.9883** |
|  |  | new_opt | 0.9927 | 0.9854 | 0.9890 | 0.9905 | 0.9856 | 0.9880 | 0.9909 | 0.9856 | 0.9882 |
| HG005 | INDEL | map_ont | 0.8215 | 0.7276 | 0.7717 | 0.8277 | **0.7348** | 0.7785 | 0.8263 | 0.7332 | 0.7769 |
|  |  | new_opt | **0.8219** | 0.7276 | **0.7719** | 0.8277 | 0.7347 | 0.7785 | **0.8264** | 0.7332 | **0.7770** |
|  | SNP | map_ont | 0.9974 | 0.9957 | 0.9965 | 0.9969 | 0.9959 | 0.9964 | 0.9970 | **0.9959** | 0.9965 |
|  |  | new_opt | 0.9974 | 0.9957 | **0.9966** | **0.9971** | 0.9959 | **0.9965** | **0.9972** | 0.9958 | 0.9965 |

**Table S2** Longshot-based SNP calling performance for Cyclone data using default and optimized mapping parameters.

| Sample | Type | Options | Training set (chr1/2/17/18) | | | Held-out set (Non-chr1/2/17/18) | | | Whole genome | | | Wall-clock time | Relative runtime |
| --- | --- | --- | --- | --- | --- | --- | --- | --- | --- | --- | --- | --- | --- |
|  |  |  | Precision | Recall | F1 score | Precision | Recall | F1 score | Precision | Recall | F1 score |  |  |
| HG002 | SNP | map_ont | 0.9797 | 0.9701 | 0.9749 | 0.9735 | 0.9645 | 0.9690 | 0.9748 | 0.9657 | 0.9702 | 15701.027 | 1.0000 |
|  |  | new_opt | 0.9797 | **0.9702** | 0.9749 | **0.9736** | 0.9645 | 0.9690 | **0.9749** | 0.9657 | **0.9703** | **5654.453** | **0.3601** |
| HG005 | SNP | map_ont | 0.9918 | 0.9885 | **0.9902** | 0.9915 | 0.9825 | 0.9870 | 0.9915 | 0.9838 | 0.9877 | 15061.693 | 1.0000 |
|  |  | new_opt | 0.9918 | 0.9885 | 0.9901 | 0.9915 | 0.9825 | 0.9870 | 0.9915 | 0.9838 | 0.9877 | **6056.582** | **0.4021** |

**Table S3** SV calling performance for Cyclone data using default and optimized mapping parameters.

| Sample | Options | Software | Training set (chr1/2/17/18) | | | Held-out set (Non-chr1/2/17/18) | | | Whole genome | | |
| --- | --- | --- | --- | --- | --- | --- | --- | --- | --- | --- | --- |
|  |  |  | Precision | Recall | F1 score | Precision | Recall | F1 score | Precision | Recall | F1 score |
| HG002 | new_opt | sniffles | **0.9388** | **0.6889** | **0.7947** | **0.9237** | **0.7011** | **0.7971** | **0.9268** | **0.6985** | **0.7966** |
|  |  | cutesv | **0.9446** | **0.6946** | **0.8006** | **0.9417** | **0.7037** | **0.8055** | **0.9423** | **0.7018** | **0.8044** |
|  | map_ont | sniffles | 0.9273 | 0.6768 | 0.7825 | 0.9152 | 0.6918 | 0.7880 | 0.9177 | 0.6886 | 0.7868 |
|  |  | cutesv | 0.9313 | 0.6943 | 0.7955 | 0.9260 | 0.7010 | 0.7980 | 0.9271 | 0.6996 | 0.7974 |
| CHM13-sim | new_opt | sniffles | **0.7952** | 0.7016 | **0.7455** | **0.6932** | 0.6817 | **0.6874** | **0.7115** | 0.6856 | **0.6983** |
|  |  | cutesv | **0.8629** | 0.6458 | **0.7387** | **0.7444** | 0.6334 | **0.6844** | **0.7653** | 0.6358 | **0.6946** |
|  | map_ont | sniffles | 0.7688 | **0.7152** | 0.7410 | 0.6658 | **0.6861** | 0.6758 | 0.6844 | **0.6918** | 0.6881 |
|  |  | cutesv | 0.8415 | **0.6494** | 0.7331 | 0.7141 | **0.6357** | 0.6726 | 0.7365 | **0.6384** | 0.6839 |

**Table S4** Coverage-dependent SV calling performance for HG002 ONT data using default and optimized minimap2 parameters.

| Depth | Options | Software | Training set (chr1/2/17/18) | | | Held-out set (Non-chr1/2/17/18) | | | Whole genome | | | Wall-clock time | Relative runtime |
| --- | --- | --- | --- | --- | --- | --- | --- | --- | --- | --- | --- | --- | --- |
|  |  |  | Precision | Recall | F1 score | Precision | Recall | F1 score | Precision | Recall | F1 score |  |  |
| 5x | Best option | sniffles | 0.9266 | **0.5877** | **0.7192** | 0.9176 | **0.5797** | **0.7106** | 0.9195 | **0.5814** | **0.7124** | **480.6460** | **0.1369** |
|  |  | cutesv | **0.9539** | **0.6003** | **0.7369** | **0.9435** | **0.6021** | **0.7351** | **0.9457** | **0.6017** | **0.7355** |  |  |
|  | lr:hq (default) | sniffles | **0.9440** | 0.5662 | 0.7078 | **0.9337** | 0.5650 | 0.7040 | **0.9359** | 0.5652 | 0.7048 | 695.0120 | 0.1979 |
|  |  | cutesv | 0.9443 | 0.5859 | 0.7231 | 0.9386 | 0.5920 | 0.7261 | 0.9398 | 0.5907 | 0.7254 |  |  |
| 10x | Best option | sniffles | 0.9213 | **0.7056** | **0.7991** | 0.9139 | **0.7077** | **0.7977** | 0.9154 | **0.7072** | **0.7980** | **851.2320** | **0.2424** |
|  |  | cutesv | **0.9560** | **0.6884** | **0.8004** | **0.9506** | **0.6844** | **0.7958** | **0.9517** | **0.6853** | **0.7968** |  |  |
|  | lr:hq (default) | sniffles | **0.9403** | 0.6840 | 0.7919 | **0.9322** | 0.6889 | 0.7923 | **0.9339** | 0.6879 | 0.7922 | 1263.9450 | 0.3599 |
|  |  | cutesv | 0.9483 | 0.6705 | 0.7856 | 0.9456 | 0.6710 | 0.7850 | 0.9462 | 0.6709 | 0.7851 |  |  |
| 15x | Best option | sniffles | 0.9336 | **0.7377** | **0.8242** | 0.9255 | **0.7368** | **0.8204** | 0.9272 | **0.7370** | **0.8212** | **1275.1790** | **0.3631** |
|  |  | cutesv | **0.9606** | **0.6818** | **0.7975** | **0.9540** | **0.6818** | **0.7952** | **0.9554** | **0.6818** | **0.7957** |  |  |
|  | lr:hq (default) | sniffles | **0.9431** | 0.7160 | 0.8140 | **0.9352** | 0.7204 | 0.8138 | **0.9368** | 0.7195 | 0.8139 | 1853.2520 | 0.5277 |
|  |  | cutesv | 0.9512 | 0.6749 | 0.7896 | 0.9492 | 0.6726 | 0.7873 | 0.9496 | 0.6731 | 0.7878 |  |  |
| 30x | Best option | sniffles | **0.9459** | **0.7465** | **0.8344** | **0.9375** | **0.7574** | **0.8378** | **0.9392** | **0.7551** | **0.8371** | **2369.3420** | **0.6747** |
|  |  | cutesv | **0.9549** | **0.7372** | **0.8321** | **0.9497** | **0.7380** | **0.8306** | **0.9508** | **0.7379** | **0.8309** |  |  |
|  | lr:hq (default) | sniffles | 0.9453 | 0.7283 | 0.8227 | 0.9372 | 0.7397 | 0.8268 | 0.9389 | 0.7373 | 0.8260 | 3511.8560 | 1.0000 |
|  |  | cutesv | 0.9494 | 0.7279 | 0.8241 | 0.9480 | 0.7268 | 0.8228 | 0.9483 | 0.7270 | 0.8230 |  |  |

**Table S5** SV calling performance for CHM13-based synthetic ONT data using default and optimized minimap2 parameters.

| Depth | Options | Software | Training set (chr1/2/17/18) | | | Held-out set (Non-chr1/2/17/18) | | | Whole genome | | |
| --- | --- | --- | --- | --- | --- | --- | --- | --- | --- | --- | --- |
|  |  |  | Precision | Recall | F1 score | Precision | Recall | F1 score | Precision | Recall | F1 score |
| 5x | Best option | sniffles | **0.8191** | **0.6939** | **0.7513** | **0.8009** | **0.6783** | **0.7345** | **0.8045** | **0.6813** | **0.7378** |
|  |  | cutesv | 0.8219 | **0.6963** | **0.7539** | **0.8092** | **0.6785** | **0.7381** | **0.8117** | **0.6820** | **0.7412** |
|  | lr:hq (default) | sniffles | 0.8175 | 0.6772 | 0.7408 | 0.7227 | 0.6606 | 0.6903 | 0.7399 | 0.6639 | 0.6998 |
|  |  | cutesv | **0.8250** | 0.6767 | 0.7436 | 0.7324 | 0.6628 | 0.6959 | 0.7493 | 0.6655 | 0.7049 |
| 10x | Best option | sniffles | 0.7637 | **0.7606** | **0.7622** | **0.7634** | **0.7336** | **0.7482** | **0.7635** | **0.7389** | **0.7510** |
|  |  | cutesv | 0.8221 | **0.7396** | **0.7787** | **0.8085** | **0.7168** | **0.7599** | **0.8112** | **0.7213** | **0.7636** |
|  | lr:hq (default) | sniffles | **0.7651** | 0.7365 | 0.7505 | 0.6917 | 0.7141 | 0.7027 | 0.7052 | 0.7185 | 0.7118 |
|  |  | cutesv | **0.8234** | 0.7275 | 0.7725 | 0.7300 | 0.6996 | 0.7145 | 0.7472 | 0.7051 | 0.7256 |
| 15x | Best option | sniffles | **0.7494** | **0.7715** | **0.7603** | **0.7518** | **0.7455** | **0.7486** | **0.7513** | **0.7506** | **0.7509** |
|  |  | cutesv | **0.8493** | **0.7270** | **0.7834** | **0.8227** | **0.7086** | **0.7614** | **0.8280** | **0.7122** | **0.7657** |
|  | lr:hq (default) | sniffles | 0.7479 | 0.7461 | 0.7470 | 0.6781 | 0.7226 | 0.6996 | 0.6910 | 0.7272 | 0.7086 |
|  |  | cutesv | 0.8457 | 0.7103 | 0.7721 | 0.7414 | 0.6924 | 0.7161 | 0.7602 | 0.6959 | 0.7267 |
| 30x | Best option | sniffles | **0.7361** | **0.7730** | **0.7541** | **0.7415** | **0.7518** | **0.7466** | **0.7404** | **0.7560** | **0.7481** |
|  |  | cutesv | **0.8434** | **0.7290** | **0.7820** | **0.8218** | **0.7135** | **0.7639** | **0.8261** | **0.7166** | **0.7674** |
|  | lr:hq (default) | sniffles | 0.7311 | 0.7546 | 0.7426 | 0.6698 | 0.7327 | 0.6998 | 0.6812 | 0.7370 | 0.7080 |
|  |  | cutesv | 0.8412 | 0.7123 | 0.7714 | 0.7342 | 0.6947 | 0.7139 | 0.7534 | 0.6981 | 0.7247 |

**Table S6** Coverage-dependent SV calling performance for HG002 HiFi data using default and optimized minimap2 parameters.

| Depth | Options | Software | Training set (chr1/2/17/18) | | | Held-out set (Non-chr1/2/17/18) | | | Whole genome | | | Wall-clock time | Relative runtime |
| --- | --- | --- | --- | --- | --- | --- | --- | --- | --- | --- | --- | --- | --- |
|  |  |  | Precision | Recall | F1 score | Precision | Recall | F1 score | Precision | Recall | F1 score |  |  |
| 5x | Best option | sniffles | **0.9586** | **0.5598** | **0.7068** | 0.9492 | **0.5471** | **0.6941** | **0.9512** | **0.5497** | **0.6968** | **442.2580** | **0.1357** |
|  |  | cutesv | **0.9545** | **0.5646** | **0.7096** | 0.9468 | **0.5615** | **0.7050** | 0.9485 | **0.5622** | **0.7059** |  |  |
|  | map_hifi (default) | sniffles | 0.9521 | 0.5421 | 0.6908 | **0.9493** | 0.5386 | 0.6873 | 0.9499 | 0.5393 | 0.6880 | 617.1340 | 0.1893 |
|  |  | cutesv | 0.9522 | 0.5519 | 0.6987 | **0.9490** | 0.5522 | 0.6981 | **0.9497** | 0.5521 | 0.6983 |  |  |
| 10x | Best option | sniffles | **0.9521** | **0.6939** | **0.8028** | **0.9465** | **0.6847** | **0.7946** | **0.9477** | **0.6866** | **0.7963** | **780.2370** | **0.2393** |
|  |  | cutesv | **0.9575** | **0.6537** | **0.7770** | 0.9525 | **0.6413** | **0.7665** | 0.9535 | **0.6439** | **0.7687** |  |  |
|  | map_hifi (default) | sniffles | 0.9475 | 0.6763 | 0.7892 | 0.9450 | 0.6742 | 0.7870 | 0.9456 | 0.6746 | 0.7874 | 1128.3030 | 0.3461 |
|  |  | cutesv | 0.9561 | 0.6367 | 0.7644 | **0.9530** | 0.6320 | 0.7600 | **0.9536** | 0.6330 | 0.7609 |  |  |
| 15x | Best option | sniffles | **0.9524** | **0.7224** | **0.8216** | **0.9462** | **0.7216** | **0.8188** | **0.9475** | **0.7218** | **0.8194** | **1163.6110** | **0.3569** |
|  |  | cutesv | **0.9655** | **0.6480** | **0.7755** | 0.9567 | **0.6288** | **0.7589** | **0.9586** | **0.6329** | **0.7624** |  |  |
|  | map_hifi (default) | sniffles | 0.9492 | 0.7047 | 0.8089 | 0.9431 | 0.7096 | 0.8099 | 0.9443 | 0.7086 | 0.8097 | 1647.1670 | 0.5052 |
|  |  | cutesv | 0.9603 | 0.6338 | 0.7636 | **0.9575** | 0.6178 | 0.7510 | 0.9581 | 0.6212 | 0.7537 |  |  |
| 30x | Best option | sniffles | 0.9470 | **0.7434** | **0.8330** | **0.9457** | **0.7445** | **0.8331** | **0.9460** | **0.7443** | **0.8331** | **2146.6020** | **0.6584** |
|  |  | cutesv | **0.9603** | **0.6899** | **0.8029** | 0.9518 | **0.6825** | **0.7949** | 0.9536 | **0.6841** | **0.7966** |  |  |
|  | map_hifi (default) | sniffles | **0.9482** | 0.7215 | 0.8195 | 0.9410 | 0.7323 | 0.8237 | 0.9425 | 0.7301 | 0.8228 | 3260.2450 | 1.0000 |
|  |  | cutesv | 0.9580 | 0.6763 | 0.7928 | **0.9533** | 0.6711 | 0.7877 | **0.9543** | 0.6722 | 0.7888 |  |  |

**Table S7** SV calling performance for CHM13-based synthetic HiFi data using default and optimized minimap2 parameters.

| Depth | Options | Software | Training set (chr1/2/17/18) | | | Held-out set (Non-chr1/2/17/18) | | | Whole genome | | |
| --- | --- | --- | --- | --- | --- | --- | --- | --- | --- | --- | --- |
|  |  |  | Precision | Recall | F1 score | Precision | Recall | F1 score | Precision | Recall | F1 score |
| 5x | Best option | sniffles | 0.8462 | **0.6153** | **0.7125** | **0.8646** | **0.6093** | **0.7149** | **0.8609** | **0.6105** | **0.7144** |
|  |  | cutesv | 0.8529 | **0.5675** | 0.6815 | 0.8694 | **0.5632** | **0.6836** | 0.8661 | **0.5640** | **0.6832** |
|  | map_hifi (default) | sniffles | **0.8632** | 0.6061 | 0.7122 | 0.8489 | 0.5962 | 0.7004 | 0.8517 | 0.5981 | 0.7027 |
|  |  | cutesv | **0.8740** | 0.5636 | **0.6853** | **0.8717** | 0.5518 | 0.6758 | **0.8721** | 0.5541 | 0.6776 |
| 10x | Best option | sniffles | 0.8178 | **0.6760** | 0.7402 | **0.8324** | **0.6652** | **0.7395** | **0.8294** | **0.6673** | **0.7396** |
|  |  | cutesv | 0.8520 | **0.6054** | **0.7078** | 0.8662 | **0.6006** | **0.7093** | 0.8633 | **0.6015** | **0.7090** |
|  | map_hifi (default) | sniffles | **0.8328** | 0.6719 | **0.7438** | 0.8179 | 0.6503 | 0.7246 | 0.8209 | 0.6546 | 0.7283 |
|  |  | cutesv | **0.8709** | 0.5943 | 0.7065 | **0.8672** | 0.5866 | 0.6998 | **0.8679** | 0.5881 | 0.7011 |
| 15x | Best option | sniffles | 0.8144 | **0.6903** | **0.7472** | **0.8212** | **0.6732** | **0.7399** | **0.8198** | **0.6765** | **0.7413** |
|  |  | cutesv | 0.8662 | **0.6030** | 0.7110 | 0.8753 | **0.5957** | **0.7089** | 0.8735 | **0.5971** | **0.7094** |
|  | map_hifi (default) | sniffles | **0.8198** | 0.6780 | 0.7422 | 0.8077 | 0.6621 | 0.7276 | 0.8101 | 0.6652 | 0.7305 |
|  |  | cutesv | **0.8861** | 0.5945 | **0.7116** | **0.8779** | 0.5831 | 0.7008 | **0.8795** | 0.5853 | 0.7029 |
| 30x | Best option | sniffles | 0.8060 | **0.7031** | **0.7510** | **0.8076** | **0.6784** | **0.7374** | **0.8073** | **0.6833** | **0.7401** |
|  |  | cutesv | 0.8585 | **0.6069** | **0.7111** | **0.8721** | **0.5974** | **0.7091** | 0.8693 | **0.5993** | **0.7095** |
|  | map_hifi (default) | sniffles | **0.8096** | 0.6934 | 0.7470 | 0.7928 | 0.6692 | 0.7258 | 0.7961 | 0.6740 | 0.7300 |
|  |  | cutesv | **0.8765** | 0.5950 | 0.7088 | 0.8702 | 0.5844 | 0.6992 | **0.8715** | 0.5865 | 0.7011 |

**Table S8** Coverage-dependent SV calling performance for HG002 Cyclone data using default and optimized minimap2 parameters.

| Depth | Options | Software | Training set (chr1/2/17/18) | | | Held-out set (Non-chr1/2/17/18) | | | Whole genome | | | Wall-clock time | Relative runtime |
| --- | --- | --- | --- | --- | --- | --- | --- | --- | --- | --- | --- | --- | --- |
|  |  |  | Precision | Recall | F1 score | Precision | Recall | F1 score | Precision | Recall | F1 score |  |  |
| 5x | Best option | sniffles | 0.8984 | **0.5668** | **0.6951** | 0.8983 | **0.5493** | **0.6817** | 0.8984 | **0.5530** | **0.6846** | **638.6830** | **0.1698** |
|  |  | cutesv | 0.9123 | **0.5779** | **0.7076** | 0.9078 | **0.5540** | **0.6881** | 0.9088 | **0.5590** | **0.6922** |  |  |
|  | Default option | sniffles | **0.9246** | 0.5519 | 0.6912 | **0.9104** | 0.5368 | 0.6754 | **0.9135** | 0.5400 | 0.6787 | 708.3230 | 0.1883 |
|  |  | cutesv | **0.9247** | 0.5497 | 0.6895 | **0.9165** | 0.5374 | 0.6776 | **0.9182** | 0.5400 | 0.6801 |  |  |
| 10x | Best option | sniffles | 0.9097 | **0.6805** | **0.7786** | 0.9020 | **0.6805** | **0.7757** | 0.9036 | **0.6805** | **0.7763** | **1226.8020** | **0.3261** |
|  |  | cutesv | 0.9334 | **0.6561** | **0.7705** | **0.9346** | **0.6378** | **0.7581** | 0.9343 | **0.6416** | **0.7608** |  |  |
|  | Default option | sniffles | **0.9290** | 0.6532 | 0.7670 | **0.9126** | 0.6569 | 0.7639 | **0.9160** | 0.6561 | 0.7646 | 1344.6330 | 0.3574 |
|  |  | cutesv | **0.9454** | 0.6286 | 0.7551 | 0.9327 | 0.6134 | 0.7401 | **0.9354** | 0.6166 | 0.7433 |  |  |
| 15x | Best option | sniffles | 0.9246 | **0.7079** | **0.8019** | 0.9156 | **0.7064** | **0.7975** | 0.9175 | **0.7067** | **0.7984** | **1793.3320** | **0.4767** |
|  |  | cutesv | 0.9486 | **0.6500** | **0.7714** | **0.9454** | **0.6315** | **0.7572** | **0.9461** | **0.6354** | **0.7602** |  |  |
|  | Default option | sniffles | **0.9361** | 0.6803 | 0.7879 | **0.9215** | 0.6828 | 0.7844 | **0.9246** | 0.6823 | 0.7852 | 1952.3460 | 0.5190 |
|  |  | cutesv | **0.9496** | 0.6303 | 0.7577 | 0.9414 | 0.6140 | 0.7433 | 0.9431 | 0.6175 | 0.7463 |  |  |
| 30x | Best option | sniffles | 0.9279 | **0.7195** | **0.8105** | 0.9224 | **0.7210** | **0.8093** | 0.9235 | **0.7207** | **0.8096** | **3454.6210** | **0.9183** |
|  |  | cutesv | **0.9453** | **0.7007** | **0.8048** | **0.9406** | **0.6887** | **0.7952** | **0.9416** | **0.6912** | **0.7972** |  |  |
|  | Default option | sniffles | **0.9336** | 0.6921 | 0.7949 | **0.9245** | 0.6996 | 0.7965 | **0.9264** | 0.6980 | 0.7961 | 3761.9850 | 1.0000 |
|  |  | cutesv | 0.9448 | 0.6734 | 0.7864 | 0.9384 | 0.6657 | 0.7789 | 0.9397 | 0.6673 | 0.7805 |  |  |

The default option represents the optimal parameters we identified for general-purpose mapping.

**Table S9** SV calling performance for CHM13-based synthetic Cyclone data using default and optimized minimap2 parameters.

| Depth | Options | Software | Training set (chr1/2/17/18) | | | Held-out set (Non-chr1/2/17/18) | | | Whole genome | | |
| --- | --- | --- | --- | --- | --- | --- | --- | --- | --- | --- | --- |
|  |  |  | Precision | Recall | F1 score | Precision | Recall | F1 score | Precision | Recall | F1 score |
| 5x | Best option | sniffles | **0.8560** | **0.6390** | **0.7318** | **0.8214** | **0.6182** | **0.7054** | **0.8281** | **0.6222** | **0.7106** |
|  |  | cutesv | 0.8270 | **0.6267** | **0.7130** | **0.8102** | **0.6169** | **0.7004** | **0.8135** | **0.6188** | **0.7029** |
|  | Default option | sniffles | 0.8509 | 0.6248 | 0.7205 | 0.7324 | 0.6090 | 0.6650 | 0.7534 | 0.6121 | 0.6754 |
|  |  | cutesv | **0.8370** | 0.6110 | 0.7063 | 0.7322 | 0.5987 | 0.6587 | 0.7510 | 0.6011 | 0.6678 |
| 10x | Best option | sniffles | 0.8129 | **0.6978** | **0.7510** | **0.7964** | **0.6734** | **0.7298** | **0.7996** | **0.6782** | **0.7339** |
|  |  | cutesv | 0.8517 | **0.6659** | **0.7474** | **0.8189** | **0.6487** | **0.7240** | **0.8252** | **0.6521** | **0.7285** |
|  | Default option | sniffles | **0.8143** | 0.6714 | 0.7360 | 0.7081 | 0.6603 | 0.6834 | 0.7270 | 0.6625 | 0.6933 |
|  |  | cutesv | **0.8534** | 0.6463 | 0.7355 | 0.7352 | 0.6347 | 0.6812 | 0.7562 | 0.6369 | 0.6915 |
| 15x | Best option | sniffles | 0.7923 | **0.6990** | 0.7427 | **0.7897** | **0.6870** | **0.7348** | **0.7902** | **0.6894** | **0.7364** |
|  |  | cutesv | 0.8643 | **0.6497** | **0.7418** | **0.8365** | **0.6456** | **0.7287** | **0.8418** | **0.6464** | **0.7312** |
|  | Default option | sniffles | **0.8027** | 0.6937 | **0.7442** | 0.6983 | 0.6737 | 0.6857 | 0.7169 | 0.6776 | 0.6967 |
|  |  | cutesv | **0.8691** | 0.6427 | 0.7389 | 0.7445 | 0.6293 | 0.6821 | 0.7665 | 0.6319 | 0.6927 |
| 30x | Best option | sniffles | 0.7918 | **0.7106** | **0.7490** | **0.7892** | **0.7006** | **0.7423** | **0.7897** | **0.7026** | **0.7436** |
|  |  | cutesv | 0.8556 | **0.6528** | **0.7406** | **0.8277** | **0.6470** | **0.7263** | **0.8331** | **0.6481** | **0.7290** |
|  | Default option | sniffles | **0.7952** | 0.7016 | 0.7455 | 0.6932 | 0.6817 | 0.6874 | 0.7115 | 0.6856 | 0.6983 |
|  |  | cutesv | **0.8629** | 0.6458 | 0.7387 | 0.7444 | 0.6334 | 0.6844 | 0.7653 | 0.6358 | 0.6946 |

The default option represents the optimal parameters we identified for general-purpose mapping.

**Table S10** Clair3-based SNP and INDEL calling performance using default and SV-optimized mapping parameters.

| Technology | Depth | Options | Type | Precision | Recall | F1 score |
| --- | --- | --- | --- | --- | --- | --- |
| Cyclone | 15x | default_opt | INDEL | 0.7400 | **0.3972** | **0.5170** |
|  |  |  | SNP | 0.9860 | **0.9688** | **0.9773** |
|  |  | new_opt | INDEL | **0.7557** | 0.3923 | 0.5165 |
|  |  |  | SNP | **0.9862** | 0.9683 | 0.9772 |
|  | 30x | default_opt | INDEL | 0.7488 | **0.4636** | 0.5727 |
|  |  |  | SNP | 0.9900 | 0.9828 | 0.9864 |
|  |  | new_opt | INDEL | **0.7602** | 0.4619 | **0.5746** |
|  |  |  | SNP | **0.9901** | **0.9842** | **0.9871** |
| HiFi | 15x | default_opt | INDEL | 0.9043 | 0.8468 | 0.8746 |
|  |  |  | SNP | 0.9916 | **0.9880** | 0.9898 |
|  |  | new_opt | INDEL | **0.9054** | **0.8476** | **0.8755** |
|  |  |  | SNP | **0.9934** | 0.9876 | **0.9905** |
|  | 30x | default_opt | INDEL | 0.9372 | 0.9177 | 0.9274 |
|  |  |  | SNP | 0.9941 | **0.9948** | 0.9944 |
|  |  | new_opt | INDEL | **0.9384** | **0.9189** | **0.9285** |
|  |  |  | SNP | **0.9955** | 0.9946 | **0.9951** |
| ONT | 15x | default_opt | INDEL | **0.5418** | **0.6553** | **0.5932** |
|  |  |  | SNP | **0.9488** | 0.9885 | **0.9683** |
|  |  | new_opt | INDEL | 0.5382 | 0.6541 | 0.5905 |
|  |  |  | SNP | 0.9457 | **0.9893** | 0.9670 |
|  | 30x | default_opt | INDEL | 0.7194 | **0.6737** | 0.6958 |
|  |  |  | SNP | **0.9934** | 0.9910 | 0.9922 |
|  |  | new_opt | INDEL | **0.7236** | 0.6719 | **0.6968** |
|  |  |  | SNP | 0.9924 | **0.9921** | 0.9922 |

**Table S11** Computational resource usage of mapping parameter optimization for Cyclone data.

| Type | Simulation data | Bayesian parameter search | Partial real-data evaluation | Whole-genome validation |
| --- | --- | --- | --- | --- |
| Wall-clock Time (h) | 0.35 | 113.50 | 9.14 | 29.35 |
| Peak RSS (GB) | 6.89 | 15.67 | 20.22 | 83.76 |

All benchmarks were performed on the same computer with 60 CPU cores and 376 GB of memory.
