# Supplementary material for: Context-aware simulation enables systematic optimization of long-read mapping parameters
Source: Gigascience. 2026 Jul 8;15:giag079. doi: 10.1093/gigascience/giag079 (PMC13401045; doi:10.1093/gigascience/giag079)
Supplement: giag079_GIGA-D-26-00085_Revision_2 [file giag079_giga-d-26-00085_revision_2.pdf]

GigaScience

Context-aware simulation enables systematic optimization of long-read mapping parameters

--Manuscript Draft--

**Manuscript Number:**	GIGA-D-26-00085R2
**Full Title:**	Context-aware simulation enables systematic optimization of long-read mapping parameters
**Article Type:**	Technical Note
**Funding Information:**	Hubei Provincial Natural Science Foundation of China (JCZRMS202600562) — Mr jiang hu
**Abstract:**	Long-read mapping performance is critical for downstream genomic analyses but remains sensitive to parameter selection. We present CycSim, a context-aware long-read simulator that learns sequence-context-dependent error profiles from empirical data and generates realistic simulated reads. CycSim more faithfully recapitulated real long-read characteristics than existing simulators, providing a high-fidelity simulation framework with known ground truth. Using this framework, we identified a Cyclone-specific parameter set that achieved 2.78-fold faster mapping than an ONT-oriented baseline while maintaining comparable variant-calling performance. For SV-oriented optimization, CycSim-guided refinement improved mapping efficiency by 8.14–34.16% across ONT, HiFi, and Cyclone HG002 datasets, increased SV F1 scores by 0.57–1.75 percentage points, and showed consistent improvements across independent benchmark datasets and different SV callers. Together, these results demonstrate the utility of CycSim for platform- and analysis-goal-specific algorithm development, benchmarking, and parameter optimization.
**Corresponding Author:**	Chentao Yang<br>BGI-Shenzhen: BGI Group<br>Shenzhen, Guangdong CHINA
**Corresponding Author Secondary Information:**	
**Corresponding Author's Institution:**	BGI-Shenzhen: BGI Group
**Corresponding Author's Secondary Institution:**	
**First Author:**	jiang hu
**First Author Secondary Information:**	
**Order of Authors:**	jiang hu<br>Dongming Fang<br>Xin Jin<br>Chentao Yang
**Order of Authors Secondary Information:**	
**Response to Reviewers:**	Dear Editor and Reviewer,<br>We sincerely thank the Editor and Reviewer for the positive assessment of our revised manuscript and for the remaining constructive suggestion. We have carefully addressed the remaining point as described below.<br>Editor comment: Please register any new software application in the bio.tools and SciCrunch.org databases to receive RRID and biotoolsID identifiers, and include these in your manuscript.<br>Response:<br>As described in the previous revision, CycSim has been registered in both bio.tools and SciCrunch. It has been assigned the biotoolsID cycsim and RRID SCR_028425. These identifiers have been included in the “Availability of Source Code and Requirements” section of the revised manuscript.

	Reviewer #1<br>Comment: The authors have addressed most of my concerns by adding Clair3-based SNP and indel evaluation. My only remaining concern is that the Clair3 model used for Cyclone data is ONT-trained, which may not fully match Cyclone error profiles. If feasible, I suggest adding a platform-matched evaluation, such as longcallD or a Clair3 model retrained/fine-tuned on Cyclone data.<br>Response:<br>We thank the reviewer for this thoughtful suggestion. We agree that a platform-matched small-variant caller would be ideal for Cyclone data. However, to our knowledge, no publicly available Cyclone-specific Clair3 model is currently available. We also contacted the Cyclone team regarding this issue, and they confirmed that no public Cyclone-specific Clair3 model is available at present and suggested using the ONT-compatible Clair3 model as the closest available alternative. Therefore, we used the ONT-compatible r941_prom_hac_g360+g422 Clair3 model for Cyclone SNP and indel calling.<br>Although this model is not Cyclone-specific, our results suggest that it provides a reasonable small-variant evaluation for the current study. First, the Clair3-based SNP and indel results on Cyclone data showed comparable high accuracy for both the optimized and baseline mapping parameters (Table S1). Second, the observed Cyclone error profiles were similar to ONT error profiles in several analyses, including substitution bias patterns and region-stratified error decomposition (Fig. 1B, Fig. S4, and Fig. S6). These results suggest that the ONT-compatible Clair3 model is unlikely to substantially bias the relative comparison between Cyclone mapping parameter sets in this study.<br>Nevertheless, we agree that a Cyclone-specific caller model would provide a more platform-matched evaluation. Retraining or fine-tuning Clair3 on Cyclone data, or evaluating with a Cyclone-validated longcallD model, would require a sufficiently curated platform-specific training set and was beyond the scope of the present revision.
**Additional Information:**	
**Question**	**Response**
Are you submitting this manuscript to a special series or article collection?	No
**Experimental design and statistics**<br><br>Full details of the experimental design and statistical methods used should be given in the Methods section, as detailed in our Minimum Standards Reporting Checklist. Information essential to interpreting the data presented should be made available in the figure legends.<br><br>Have you included all the information requested in your manuscript?	Yes
**Resources**<br><br>A description of all resources used, including antibodies, cell lines, animals and software tools, with enough information to allow them to be uniquely	Yes

identified, should be included in the Methods section. Authors are strongly encouraged to cite Research Resource Identifiers (RRIDs) for antibodies, model organisms and tools, where possible.<br><br>Have you included the information requested as detailed in our Minimum Standards Reporting Checklist?	
**Availability of data and materials**<br><br>All datasets and code on which the conclusions of the paper rely must be either included in your submission or deposited in publicly available repositories (where available and ethically appropriate), referencing such data using a unique identifier in the references and in the “Availability of Data and Materials” section of your manuscript.<br><br>Have you have met the above requirement as detailed in our Minimum Standards Reporting Checklist?	Yes
GigaScience has policies and guidelines in place for the use of generative AI-writing tools such as ChatGPT. If you have used such writing tools to assist with writing the manuscript this must be declared and cited in the text. Authors should not list AI-writing tools and other AI-assisted technologies as an author or co-author and should acknowledge that they are fully responsible for text generated or refined by AI-writing tools.<p><br><br>A summary of use (particularly in the introduction or among methods) needs to be included at the end of the paper, and the outputs should also be included as a supplementary file hosted in GigaDB or other open repositories. Please read our guidelines for more information. <p><br><br>By submitting to GigaScience, you are aware of the journal's AI-writing tools policy, and if you have declared use of	No

such tools below, you have acknowledged this where appropriate in your manuscript and have made a summary of use and outputs available. <p><br><b>AI-assisted writing tools have been used in the preparation of this manuscript?	

Manuscript

Click here to access/download;Manuscript;manuscript-giga-v3.docx

Context-aware simulation enables systematic optimization of long-read mapping parameters

Jiang Hu[1,2,3], Dongming Fang[2], Xin Jin[2], Chentao Yang[2,4,*]

1. BGI Research, Wuhan 430074, China

2. State Key Laboratory of Genome and Multi-omics Technologies, BGI Research, Shenzhen 518083, China

3. Center for Evolutionary Biology, School of Life Sciences, Fudan University, Shanghai 200438, China

4. Guangdong Provincial Key Laboratory of Genome Read and Write, BGI Research, Shenzhen 518083, China

*Corresponding authors: Chentao Yang: yangchentao@genomics.cn

Jiang Hu [0000-0002-8521-9161]; Dongming Fang [0000-0002-9205-350X]; Xin Jin [0000-0001-7554-4975]; Chentao Yang [0000-0003-3447-2316]

Abstract

Long-read mapping performance is critical for downstream genomic analyses but remains sensitive to parameter selection. We present CycSim, a context-aware long-read simulator that learns sequence-context-dependent error profiles from empirical data and generates realistic simulated reads. CycSim more faithfully recapitulated real long-read characteristics than existing simulators, providing a high-fidelity simulation framework with known ground truth. Using this framework, we identified a Cyclone-specific parameter set that achieved

2.78-fold faster mapping than an ONT-oriented baseline while maintaining comparable variant-calling performance. For SV-oriented optimization, CycSim-guided refinement improved mapping efficiency by 8.14–34.16% across ONT, HiFi, and Cyclone HG002 datasets, increased SV F1 scores by 0.57–1.75 percentage points, and showed consistent improvements across independent benchmark datasets and different SV callers. Together, these results demonstrate the utility of CycSim for platform- and analysis-goal-specific algorithm development, benchmarking, and parameter optimization.

Keywords

Long-read sequencing, Long-read simulator, Context-aware simulation, Parameter tuning, Bayesian optimization

Introduction

The accuracy of long-read sequence mapping is fundamental to genomic analysis but is sensitive to parameterization [1–3]. Default parameters offer convenience but are often suboptimal, failing to generalize across sequencing platforms such as PacBio high-fidelity [4] (HiFi), Oxford Nanopore Technologies [5] (ONT), and Cyclone [6], or across analytical objectives such as structural variant (SV) detection [7]. Systematic optimization is impeded by the empirical nature of default parameters, the scarcity of datasets with known ground-truth alignments, and the lack of a

comprehensive framework for identifying optimal settings for specific data types or analyses. Simulation-based evaluation offers a practical solution, yet existing long-read simulators largely introduce errors at random, failing to capture sequence-context-dependent biases and global error rate heterogeneity characteristic of real data [8–10]. As a result, current simulators provide limited realism for benchmarking and parameter tuning.

To address this, we developed CycSim, a context-aware long-read simulator that generates reads based on K-mer contexts and error distributions learned from empirical datasets. We integrated CycSim with a Bayesian optimization framework to systematically identify optimal mapping parameters. Benchmarking confirms that CycSim reconstructs error profiles with greater realism than state-of-the-art alternatives. Furthermore, our optimization framework identified superior parameter sets for the Cyclone platform and confirmed the robustness of default settings for HiFi and ONT data. Notably, SV-specific tuning improves both alignment speeds and SV detection accuracy (F1 scores) across all three platforms.

Results

Context-aware long-read simulation

CycSim operates through a dual-stage framework comprising model

training and read simulation (**Fig. S1**). During the training stage, the algorithm characterizes read structure, including strand orientation, chimerism, aligned/unaligned lengths, and alignment identity by interrogating high-confidence alignments from input BAM files. To mitigate artifacts arising from alignment heuristics, aligned regions are re-aligned using edlib [11] and low-confidence termini are trimmed. Crucially, we model error characteristics at two complementary levels: (1) K-mer-based error modeling, which captures the empirical frequency of context-dependent substitution, insertion, and deletion via a sliding window approach; and (2) error transition modeling, which estimates the transition probabilities between consecutive error states to reflect the local continuity of error types.

In the simulation stage, these models define the genomic origin, structural composition, and expected error rate of synthetic reads. The aligned core is generated through a base-wise sliding process, in which errors are sampled according to the K-mer specific models and the empirical error transition matrix. Subsequently, Phred-scaled quality scores are assigned, and unaligned regions are appended to form complete reads. When the simulated error rate deviates markedly from the expected value, resampling is performed, and chimeric reads are constructed by concatenating independent simulated fragments.

To validate the framework, we benchmarked CycSim against BadRead [10], NanoSim [8], and PbSim3 [9] using the diploid HG002 genome [12] (Chromosomes 1, 2, 17, and 18) across ONT, HiFi and Cyclone platforms. CycSim consistently demonstrated superior fidelity in reproducing both read length and global error rate distributions (**Fig. 1A**, **Fig. S2**). Our assessment focused on three key metrics: (1) Base substitution profiles: CycSim and BadRead were the only tools to accurately capture empirically observed substitution biases (**Fig. 1B**); (2) K-mer distribution: CycSim faithfully reproduced the global K-mer frequency landscape; Specifically, it achieved the highest concordance for erroneous K-mers in Cyclone and HiFi data, while performing competitively on ONT (**Fig. 1C**). For the Raw comparison, real reads were randomly split into two subsets and compared against each other. Because both subsets originated from the same empirical sequencing dataset, their similarity represents an empirical upper bound for the expected concordance between simulated and real reads under finite sampling. (3) Error rates in simple repeats: Notably, real Cyclone and ONT data exhibit substantial error rate heterogeneity in low-complexity regions. While other simulators produced artifactually uniform positional identity profiles, CycSim more accurately recapitulated the regional heterogeneity observed in real reads, including higher alignment identity in regions with lower STR/SSR

density (**Fig. 1D, Fig. S3**). Error decomposition and region-stratified substitution analysis further showed that the low-complexity-associated error increase in real Cyclone and ONT data was mainly driven by elevated deletion rates, and that CycSim recapitulated these profiles across sequence-complexity classes (**Figs. S4-S6**). In addition, three independent CycSim simulations using the same trained model and settings produced nearly overlapping mapping identity distributions and highly consistent substitution spectra, supporting the reproducibility of CycSim-generated read profiles (**Fig. S7**). Finally, applying the HG002-trained CycSim model to an independent HG005 Cyclone dataset yielded simulated reads with alignment identity distributions and substitution profiles similar to real HG005 reads, supporting model generalization beyond the training sample (**Fig. S8**).

Collectively, these analyses demonstrate that CycSim provides a high-fidelity, platform-consistent representation of long-read characteristics, capturing both global and localized, context-dependent error biases that are insufficiently modeled by existing simulators. Nevertheless, CycSim is expected to perform best when the training data are generated from the same or a closely related genome, sequencing platform, chemistry, and library preparation protocol as the intended simulation target.

Bayesian optimization of mapping parameters

High-fidelity simulation is a prerequisite for simulation-guided optimization because an optimizer may otherwise overfit to simulator-specific artifacts, such as artificially uniform error distributions or missing sequence-context-dependent biases. Leveraging the improved realism of CycSim-generated reads, we established a four-stage Bayesian optimization framework to systematically identify optimal mapping parameters. The workflow proceeds as follows: (1) Simulation-based initialization using CycSim-generated reads with known ground-truth coordinates; (2) Bayesian parameter search via Optuna [13], which explores thousands of minimap2 [2] configurations to maximize a defined objective function; (3) Empirical screening, where top-performing parameter sets are evaluated on real data subsets; (4) Whole-genome validation to ensure robustness across full-scale datasets.

We first applied the framework to optimize general-purpose alignment (**Fig. 2A**). The optimization utilized 5× CycSim-simulated HG002 data (Chr 1, 2, 17, 18) to maximize a composite accuracy metric integrating both base-level identity and interval-level overlap. From this search, the top 40 configurations were screened using 30× real HG002 data on the same chromosomal subset, assessed using standard SNP, Indel and SV

benchmarks. The optimal configuration was subsequently validated on 44× whole-genome data.

When applied to the emerging Cyclone platform, using minimap2's ONT-oriented map-ont preset as an initial baseline, our framework identified a novel parameter set that increased mapping speed by 2.78-fold while maintaining comparable small-variant calling accuracy and modestly improving SV detection (**Figs. 2B and 2C, Tables S1-S3**). Importantly, the parameter search was performed only on HG002 chromosomes 1, 2, 17, and 18, whereas generalization was evaluated on held-out HG002 chromosomes excluding chromosomes 1, 2, 17, and 18, whole-genome HG002 data. For small variants, independent-sample validation was further performed using an HG005 dataset, whereas SV-oriented validation was extended using an independent CHM13-based synthetic SV benchmark. Small-variant accuracy was assessed using the deep-learning-based caller Clair3 [14] for SNP and Indel evaluation, together with the haplotype-aware statistical caller Longshot [15] for independent SNP validation. Across these validation settings, the optimized parameters maintained comparable SNP/Indel accuracy while substantially reducing mapping runtime and improving SV F1 scores with both Sniffles2 [16] and cuteSV [17], two widely used long-read SV callers (**Tables S1-S3**). Conversely, for HiFi and ONT

datasets, the framework identified configurations with performance close to the established minimap2 default presets. Because these presets have already been extensively optimized for mature long-read platforms, only marginal accuracy gains (<0.1% in simulation) with slightly reduced runtime were observed (**Fig. S9**). These results indicate that the framework can recover parameter settings comparable to developer-optimized defaults when existing presets are already well matched to the data, while still enabling parameter refinement for datasets with distinct read characteristics.

We next adapted the framework specifically for SV detection, for which breakpoint placement and interval-level alignment consistency are critical (**Fig. 2D**). Using the same training and validation design described above, we modified the optimization objective to maximize the SV F1 score and minimize mapping runtime and evaluated the resulting parameter sets with Sniffles2 and cuteSV.

Across ONT, HiFi, and Cyclone datasets, SV-oriented optimization yielded more efficient mapping configurations while preserving or improving SV calling accuracy. On whole-genome HG002 datasets at varying depths, and across both Sniffles2 and cuteSV, the optimized parameters achieved 30.84–32.65% faster mapping and 0.57–1.12

percentage-point higher SV F1 scores for ONT data, 28.34–34.16% faster mapping and 0.77–1.03 percentage-point higher SV F1 scores for HiFi data, and 8.14–9.83% faster mapping and 0.59–1.75 percentage-point higher SV F1 scores for Cyclone data relative to the corresponding default or baseline settings (**Figs. 2E-2H and S10, Tables S4-S9**).

Validation on held-out HG002 chromosomes excluding chromosomes 1, 2, 17, and 18, together with validation on an independent CHM13-based synthetic SV benchmark, showed the same overall trend across platforms, coverage depths, and both SV callers (**Tables S4-S9)**. These results indicate that the SV-oriented parameters were not specific to the HG002 training chromosomes, the HG002 sample, or a single downstream SV-calling method. Together, these findings support the utility of CycSim-guided optimization for deriving task-specific mapping parameters that improve SV analysis while reducing computational cost.

Discussion

Several practical considerations and limitations should be noted. First, when simulated depth substantially exceeds training depth, learned error contexts may be over-represented because k-mer-specific error models are estimated from a finite training set. CycSim provides a user-defined parameter to introduce random errors according to the global error-rate

distribution, increasing error-pattern diversity at high simulated coverage. For k-mer contexts absent from the training data, CycSim automatically falls back to the global average error model. These strategies preserve the overall error level, although some fine-scale context-specific biases may still be missed.

Second, the optimized parameters are task-specific. SV-oriented parameters improved SV calling and reduced mapping time, but they did not consistently improve SNP and indel calling across all settings (**Table S10**). This trade-off underscores the alignment heuristics where parameters relaxed to capture large structural disruptions can occasionally sacrifice single-base resolution. To facilitate practical applications, we propose a Best Practice Guideline for parameter selection: for large-scale sequencing cohorts where structural variant screening is the primary objective, the SV-optimized preset provides optimal throughput and sensitivity; conversely, for comprehensive pipelines targeting a full spectrum of genomic variations (including SNPs and Indels), the CycSim-derived general-purpose preset should be prioritized to ensure well-balanced accuracy across all variant scales.

Third, optimized parameters should be selected according to the application scenario. Minimap2 default presets remain strong

general-purpose choices, especially for mature ONT and HiFi datasets. However, they may be less optimal for emerging platforms, datasets with distinct read characteristics, or analysis-specific goals such as SV detection. In these settings, parameter refinement can improve the trade-off between downstream performance and computational efficiency. Although the SV F1-score gains were modest, the runtime reductions were substantial, making the SV-oriented parameters valuable for population-scale long-read projects where per-sample speedups accumulate across large cohorts.

Fourth, the full optimization process is computationally intensive and is therefore most suitable for large-scale or repeatedly used workflows, where the upfront search cost can be amortized across many samples (**Table S11**), or for software developers aiming to establish optimized default parameters for specific platforms and analysis tasks. Finally, the optimization framework is modular and can in principle incorporate reads generated by other simulators. Because simulation-guided optimization depends on how well simulated reads reflect real read characteristics, parameters selected from any simulator should still undergo empirical screening, independent validation, and, where necessary, additional diagnostic evaluation to avoid simulator-specific biases. More broadly, this framework provides a reproducible strategy for analysis-goal-driven

parameter refinement by combining simulation-guided candidate generation with real-data validation.

Conclusions

We have developed CycSim, a context-aware simulator that faithfully reproduces the complex error characteristics of long-read sequencing data. We paired this with an analysis-goal-driven Bayesian optimization framework that enables systematic refinement of mapping parameters. Together, these tools provide a robust foundation for improving the accuracy and efficiency of long-read analyses and optimizing bioinformatics workflows for specific platforms and analytical goals.

Methods

Model training

Reads shorter than 10 kb were removed using fxTools (v0.3.1) [18]. The remaining reads from ONT, HiFi, and Cyclone platforms were aligned to the diploid HG002 reference genome using minimap2 (v2.29) with platform-specific presets: ONT (-x lr:hq), HiFi (-x map-hifi), and Cyclone (-k16 -w13 -A2 -B4 -O4,41 -E2,1 -s180 -U70,1000000). Eight Chromosomes (1, 2, 17, and 18 from both haplotypes) and their corresponding reads were extracted for platform-specific model training using different simulators.

For CycSim, ONT and Cyclone reads models were trained using cycsim train -r nanopore -t 30 reads.bam Chr1_2_17_18.fa, while HiFi reads models used cycsim train -r hifi -t 30 reads.bam Chr1_2_17_18.fa. NanoSim (v3.2.3) models were trained with read_analysis.py genome --fastq reads.fastq.gz -rg Chr1_2_17_18.fa -t 30 -c. For Badread (v0.4.1), error and quality score models were generated using badread error_model --reference Chr1_2_17_18.fa --reads reads.fastq.gz --alignment map.paf and badread qscore_model --reference Chr1_2_17_18.fa --reads reads.fastq.gz --alignment map.paf. As PBSIM3 does not provide a training module, the pretrained models supplied by the authors for ONT and HiFi data were used directly.

Data simulation and evaluation

Simulated reads were generated at 20× depth for the same eight HG002 chromosomes using each simulator with its corresponding trained model. CycSim reads were produced using cycsim sim -t 30 -d 20 -c model.cy Chr1_2_17_18.fa. NanoSim reads were simulated with simulator.py genome -rg Chr1_2_17_18.fa -c training -x 20 -t 30 --fastq. For Badread, Cyclone reads were generated using badread simulate --reference Chr1_2_17_18.fa --quantity 20x --error_model badread_errors --qscore_model badread_qscore --length 20000,15000 --identity 97,100,2.5. ONT simulations used an adjusted identity range (20, 3), and HiFi simulations used platform-specific length (15374, 13000) and

identity (30, 3) settings. For PBSIM3, ONT reads were produced using pbsim --strategy wgs --method errhmm --errhmm ERRHMM-ONT-HQ.model --depth 20 --genome Chr1_2_17_18.fa --length-mean 20000 --accuracy-mean 0.994 --accuracy-min 0.95 --length-min 5000. HiFi reads were generated with pbsim --strategy wgs --method errhmm --errhmm ERRHMM-SEQUEL.model --depth 20 --genome Chr1_2_17_18.fa --length-mean 15374 --pass-num 15, followed by CCS processing (ccs, default parameters) to produce final HiFi reads. All simulated reads were then aligned to the reference genome using minimap2, and we quantified read length, alignment identity, and error biases for each simulator.

Alignment parameter optimization

CycSim was used to simulate reads with default settings and platform-specific trained models. Simulated ONT, HiFi, and Cyclone datasets were aligned to the reference genome using minimap2. SVs were called using Sniffles2 (v2.6.3) with the --tandem-repeats option, with additional validation using cuteSV (v2.1.3), and benchmarked against the HG002 GIAB v1.1 truth set [12] using truvari [19] with --passonly -r 1000 --refine. SNP calling was first performed with Longshot, and evaluated using hap.py with the --engine vcfeval [20]. SNPs and indels were also called with Clair3 (v2.0.0) using default parameters. For Cyclone data, the r941_prom_hac_g360+g422 model was used because

no publicly available Cyclone-specific Clair3 model was available.

To provide an additional independent validation dataset while retaining real-read characteristics as much as possible, we constructed a CHM13-based synthetic SV benchmark. HG002-derived SV alleles and sequences were incorporated into the CHM13 reference to generate a variant-integrated reference genome. Real CHM13 HiFi reads, together with ONT Q20 and Cyclone reads simulated from CHM13 by CycSim using default parameters, were aligned to the variant-integrated reference. Because the reads were derived from the original CHM13 genome, reciprocal variants between CHM13 and the variant-integrated reference were used as the synthetic truth set. SVs were called with Sniffles2 and cuteSV and benchmarked using Truvari.

For simulation-based parameter search, each minimap2 configuration was evaluated against the known truth alignment of simulated reads. Only primary alignments were retained. Two metrics were calculated: interval accuracy, defined as the fraction of reads whose predicted reference interval matched the truth interval within 50 bp, and CIGAR-operation accuracy, defined as the fraction of comparable read positions with identical CIGAR-derived operation labels between the predicted and truth alignments. The general-purpose mapping score was calculated as the

average of interval accuracy and CIGAR-operation accuracy, and Optuna minimized one minus this score. Mapping runtime was recorded for each configuration and used during empirical screening. The minimap2 search space included -k = 15 – 19, -w = 10 – 19, -A = 1 – 2, -B = 3 – 9, -O = 4 – 14,15 – 49, -E = 2 – 3,1, -s ∈ {30, 40, 80, 100, 150, 180, 200, 240}, and -U = 10 – 80,{500, 5000, 50000, 500000, 1000000}. After optimization, the top 40 non-redundant configurations were re-evaluated on 30× real HG002 data from chromosomes 1, 2, 17, and 18 using downstream SNP, Indel, and SV benchmarks. For SV-oriented optimization, the same minimap2 search space was used, but each candidate configuration was evaluated directly using downstream SV-calling performance. For each configuration, SVs were called with Sniffles2 and benchmarked with Truvari, and Optuna minimized one minus the SV F1 score. cuteSV was used for independent caller validation.

The optimal minimap2 parameters identified for SV calling were selected based on improved F1 score and reduced mapping time. For ONT data, the optimized parameters were -k 21 -w 21 -A 1 -B 9 -O 13,44 -E 3,1 -s 30 -U 20,50000. For HiFi data, the optimized SV-calling parameters were -k 23 -w 22 -A 1 -B 9 -O 13,41 -E 3,1 -s 180 -U 10,5000. For Cyclone data, the optimal parameters for general alignment were -k 16 -w 13 -A 2

-B 4 -O 4,41 -E 2,1 -s 180 -U 70,1000000, which substantially reduced mapping time while maintaining comparable alignment accuracy. In contrast, the Cyclone parameters optimized specifically for SV calling were -k 17 -w 13 -A 1 -B 9 -O 13,44 -E 3,1 -s 30 -U 70,500, which achieved a higher SV F1 score together with faster mapping.

Declarations

Ethics approval and consent to participate

Not applicable.

Consent for publication

Not applicable.

Availability of Source Code and Requirements

- Project name: CycSim
- Project home page: https://github.com/BioEarthDigital/CycSim
- Operating system(s): Linux
- Programming language: Rust
- License: MIT
- RRID：SCR_028425
- Biotools：cycsim

Data Availability

The HG002 v1.1 reference genome and HiFi reads were obtained from the HG002 repository [21]. The HG002 ONT Q20 dataset was

downloaded from the ONT Open Datasets portal [22]. HG002 Cyclone reads were retrieved from the China National GeneBank (CNGB) under accession CNP0007646, whereas the HG005 Cyclone reads were obtained from the official Cyclone team upon request. The CHM13 reference genome and HiFi reads were downloaded from the CHM13 repository [23]. The HG002 small-variant and structural-variant benchmark sets, together with the HG005 small-variant benchmark set, were downloaded from Genome in a Bottle (GIAB). CycSim and its pretrained models are released under the Massachusetts Institute of Technology (MIT) License and are available on GitHub [24] and Zenodo [25].

Competing interests

The authors are affiliated with BGI, the institutional sponsor of GigaScience. All editorial handling and peer review for this manuscript were conducted independently by the journal’s editorial team, in accordance with the journal's standard editorial policies and procedures, to ensure transparency, objectivity, and impartiality. The authors have no additional competing interests to declare.

Funding

This work was supported by Hubei Provincial Natural Science Foundation of China [grant numbers: 2026AFB569] for Jiang Hu, and

grants from the National Key R&D Program of China (2025YFC3410300) for Chentao Yang.

Authors' contributions

J.H. and C.Y. jointly designed and supervised the project. J.H. developed CycSim and drafted the manuscript. D.F. and X.J. reviewed the code and provided suggestions. All authors contributed to manuscript revision and approved the final version.

Acknowledgements

We thank Tao Zeng, Huiyuan Hao, and Jiayuan Zhang for providing the HG002 and HG005 Cyclone data.

References

1. Ayad LA, Chikhi R, Pissis SP. Seedability: optimizing alignment parameters for sensitive sequence comparison. *Bioinformatics Advances* 2023;**3**(1):vbad108. http://dx.doi.org/10.1093/bioadv/vbad108.

2. Li H. New strategies to improve minimap2 alignment accuracy. *Bioinformatics* 2021;**37**(23):4572–4. http://dx.doi.org/10.1093/bioinformatics/btab705.

3. Gamaarachchi H, Parameswaran S, Smith MA. Featherweight long

read alignment using partitioned reference indexes. *Scientific Reports* 2019;**9**(1):4318. http://dx.doi.org/10.1038/s41598-019-40739-8.

4. Eid J, Fehr A, Gray J *et al.* Real-time DNA sequencing from single polymerase molecules. *Science* 2009;**323**(5910):133–8. http://dx.doi.org/10.1126/science.1162986.

5. Branton D, Deamer DW, Marziali A *et al.* The potential and challenges of nanopore sequencing. *Nature Biotechnology* 2008;**26**(10):1146–53. http://dx.doi.org/10.1038/nbt.1495.

6. Zhang JY, Zhang Y, Wang L *et al.* A single-molecule nanopore sequencing platform. *bioRxiv* 2024:2024.08. 19.608720. http://dx.doi.org/10.1101/2024.08.19.608720.

7. Gaitán N, Duitama J. A graph clustering algorithm for detection and genotyping of structural variants from long reads. *GigaScience* 2024;**13**:giad112. http://dx.doi.org/10.1093/gigascience/giad112.

8. Yang C, Chu J, Warren RL *et al.* NanoSim: nanopore sequence read simulator based on statistical characterization. *GigaScience* 2017;**6**(4):gix010. http://dx.doi.org/10.1093/gigascience/gix010.

9. Ono Y, Hamada M, Asai K. PBSIM3: a simulator for all types of PacBio and ONT long reads. *NAR Genomics and Bioinformatics* 2022;**4**(4):lqac092. http://dx.doi.org/10.1093/nargab/lqac092.

10. Wick RR. Badread: simulation of error-prone long reads. *Journal of Open Source Software* 2019;**4**(36):1316. http://dx.doi.org/10.21105/joss.01316.

11. Šošić M, Šikić M. Edlib: a C/C++ library for fast, exact sequence alignment using edit distance. *Bioinformatics* 2017;**33**(9):1394–5. http://dx.doi.org/10.1093/bioinformatics/btw753.

12. Hansen NF, Dwarshuis N, Ji HJ *et al.* A complete diploid human genome benchmark for personalized genomics. *bioRxiv* 2025:2025.09.21.677443. http://dx.doi.org/10.1101/2025.09.21.677443.

13. Akiba T, Sano S, Yanase T *et al.* Optuna: A next-generation hyperparameter optimization framework. *Proceedings of the 25th ACM SIGKDD International Conference on Knowledge Discovery & Data Mining* 2019:2623–31. http://dx.doi.org/10.1145/3292500.3330701.

14. Zheng Z, Li S, Su J *et al.* Symphonizing pileup and full-alignment for deep learning-based long-read variant calling. *Nature Computational Science* 2022;**2**(12):797–803. http://dx.doi.org/10.1038/s43588-022-00387-x.

15. Edge P, Bansal V. Longshot enables accurate variant calling in diploid genomes from single-molecule long read sequencing. *Nature*

Communications 2019;**10**(1):4660. http://dx.doi.org/10.1038/s41467-019-12493-y.

16. Smolka M, Paulin LF, Grochowski CM *et al.* Detection of mosaic and population-level structural variants with Sniffles2. *Nature Biotechnology* 2024;**42**(10):1571–80. http://dx.doi.org/10.1038/s41587-023-02024-y.

17. Jiang T, Liu Y, Jiang Y *et al.* Long-read-based human genomic structural variation detection with cuteSV. *Genome Biol* 2020;**21**(1):189. https://doi.org/10.1186/s13059-020-02107-y.

18. Moold. fxTools: A toolset for processing sequences in FASTA/Q formats. https://github.com/moold/fxTools. Accessed 6 Jul 2026.

19. English AC, Menon VK, Gibbs RA *et al.* Truvari: refined structural variant comparison preserves allelic diversity. *Genome Biology* 2022;**23**(1):271. http://dx.doi.org/10.1186/s13059-022-02840-6.

20. Illumina. hap.py: Haplotype VCF comparison tools. https://github.com/Illumina/hap.py. Accessed 6 Jul 2026.

21. Telomere-to-Telomere Consortium. HG002: A complete diploid human genome. https://github.com/marbl/HG002. Accessed 6 Jul 2026.

22. Oxford Nanopore Technologies. GM24385 2023.12 all_pass.vhg002v1.bam. https://ont-open-data.s3.amazonaws.com/gm24385_2023.12/all_pass.vhg002v1.bam. Accessed 6 Jul 2026.

23. Telomere-to-Telomere Consortium. CHM13: The complete sequence of a human genome. https://github.com/marbl/CHM13. Accessed 6 Jul 2026.

24. Hu J. CycSim GitHub repository. https://github.com/BioEarthDigital/CycSim. Accessed 6 Jul 2026.

25. Hu J. CycSim source code in Zenodo. https://zenodo.org/records/17809771. Accessed 6 Jul 2026.

Figures

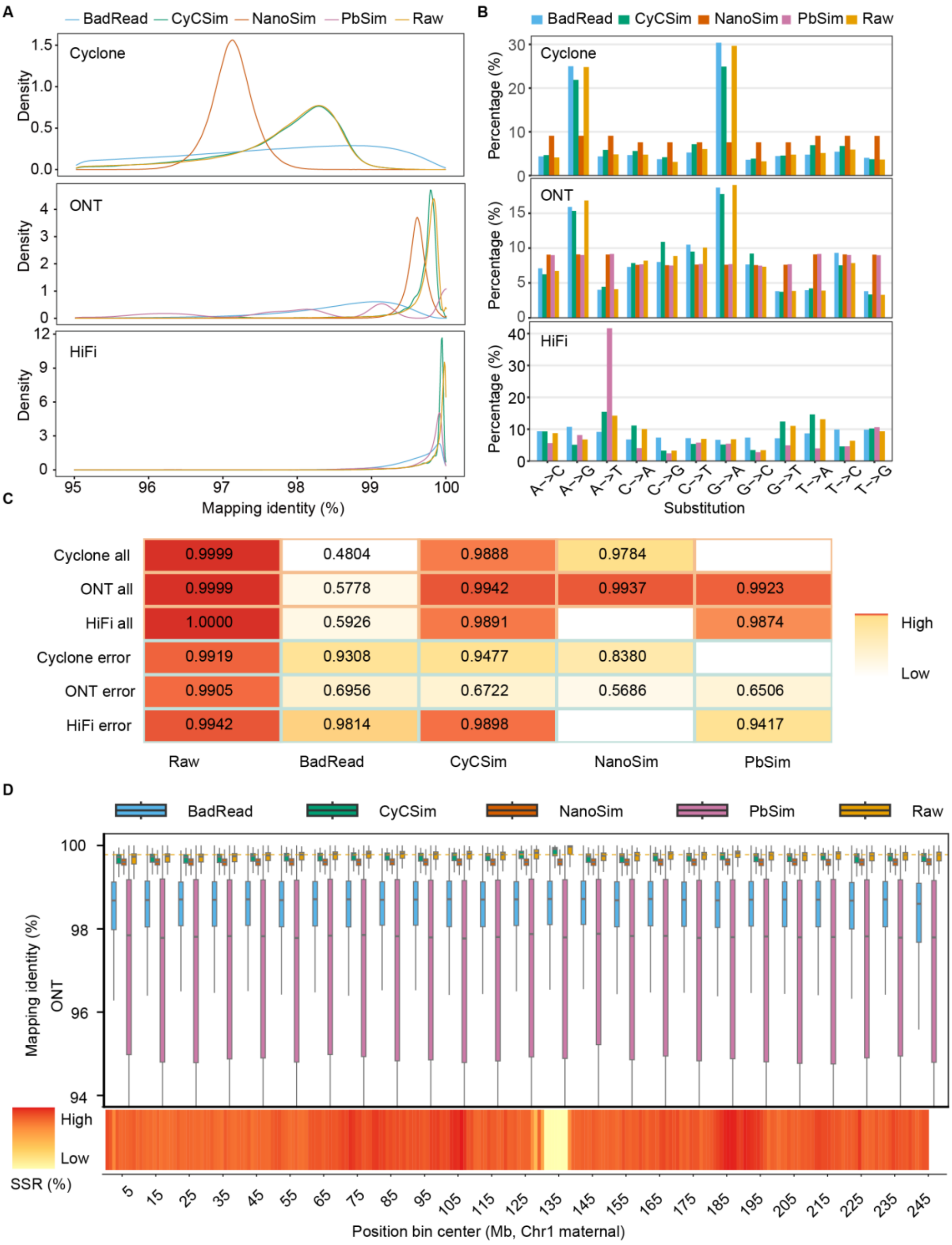

Figure 1 Multidimensional evaluation of long-read simulators. **A** Distribution of alignment identity for simulated reads compared with real reads (Raw). **B** Statistics of substitution error bias of long-read simulators. **C** Cosine similarity of K-mer count between simulated and real reads. All

denotes similarity computed over all K-mers; Error denotes K-mers present in the real reads but absent from the reference genome, representing sequencing-induced errors. For Raw, real reads were randomly split into two parts to estimate an empirical upper bound of similarity. Blank entries indicate missing values. **D** Positional alignment identity distribution along Chr1 maternal for simulated ONT reads and real reads. Horizontal yellow lines mark the median identity of real ONT reads. The lower heatmap shows the short tandem repeats (STRs) density (1–6 bp motifs, ≥3 repeat units) along Chr1 maternal. Note that genomic regions experiencing a localized dip or fragmentation in actual STR density naturally correspond to elevated empirical and simulated mapping identities, demonstrating CycSim's capacity to capture fine-grained positional heterogeneity faithfully.

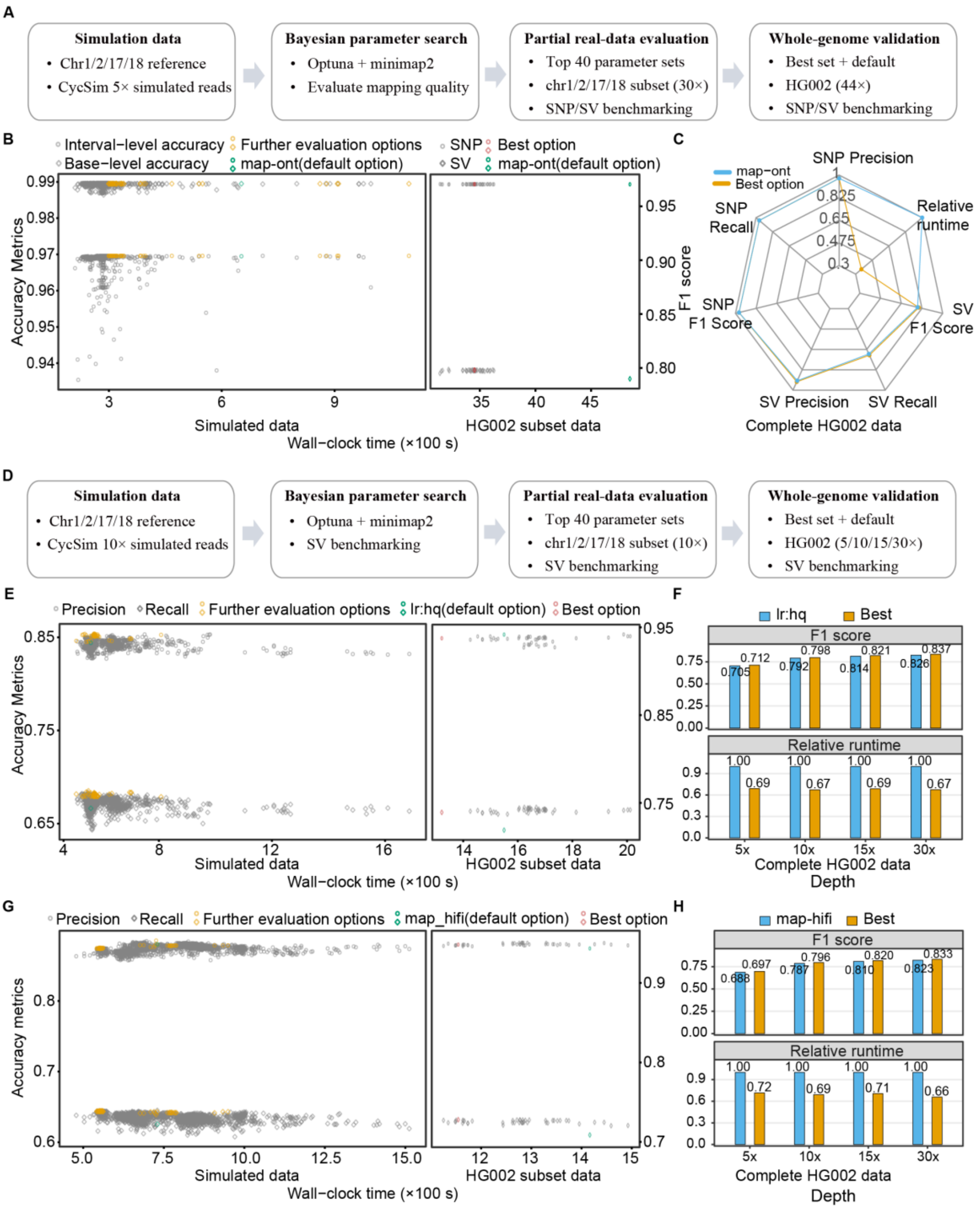

Figure 2 Bayesian optimization and evaluation of mapping parameters.

A Optimization workflow for general-purpose mapping parameters. **B** Performance of Cyclone mapping parameters on simulated and partial real reads. Interval-level accuracy denotes the proportion of aligned intervals within 50 bp of the true interval, and Base-level accuracy

denotes the proportion of correctly aligned bases. SNP and SV accuracy were assessed as described in Methods. **C** Performance of optimized versus default parameters on 44× real Cyclone reads. **D** Optimization workflow for SV detection-oriented mapping parameters using sniffles. **E** Performance of ONT SV detection-oriented mapping parameters on simulated and partial real reads. **F** Performance of optimized versus default ONT mapping parameters for SV detection at different coverage depths. **G** Performance of HiFi SV detection-oriented mapping parameters on simulated and partial real reads. **H** Performance of optimized versus default HiFi mapping parameters for SV detection at different coverage depths. For Panels B, E, and G, parameters evaluated in the right panel were selected from the left panel (labeled as Further evaluation options), and Wall-clock time includes both minimap2 and samtools sort. Relative runtime for the bar charts (Panels F and H) denotes the proportion of runtime relative to the longest run, considering minimap2 only; for the radar chart (Panel C), relative runtime is mapped as an independent axis. In all cases, lower runtime values indicate faster mapping.

Supplementary Files

Click here to access/download
Supplementary Material
supplemental_file.docx
