# Supplementary material for: Context-aware simulation enables systematic optimization of long-read mapping parameters
Source: Gigascience. 2026 Jul 8;15:giag079. doi: 10.1093/gigascience/giag079 (PMC13401045; doi:10.1093/gigascience/giag079)
Supplement: giag079_Authors_Response_To_Reviewer_Comments_Original_Submission [file giag079_authors_response_to_reviewer_comments_original_submission.pdf]

Author's Response To Reviewer Comments

Dear Editor and Reviewers,

We sincerely thank the editor and reviewers for their insightful comments and constructive suggestions. We have substantially revised the manuscript in response to these comments point by point. In particular, we have added Clair3-based SNP and indel evaluation, independent validation using an HG005 dataset and a CHM13-based synthetic SV benchmark, reproducibility analysis based on three independent CycSim simulations, region-stratified error decomposition in low-complexity regions, an explanation of why high-fidelity simulation is important for simulation-guided parameter optimization, and detailed methodological descriptions of the optimization objective, Optuna search space, benchmarking workflow, and computational cost.

We thank the Editor for this critical guidance for promoting FAIR data practices. Following your instructions, we have successfully registered CycSim in both databases. It has been assigned the bioruby/biotools ID: cycsim and RRID: SCR_028425. These identifiers, along with the WorkflowHub registration, have been explicitly integrated into the "Availability of Source Code" section of the revised manuscript.

All changes in the revised manuscript have been highlighted, and line numbers have been added.

Below, we provide a point-by-point response to the reviewers' comments.

Reviewer reports:

Reviewer #1: The authors present CycSim, a context-aware long-read simulator, and a Bayesian optimization framework designed to systematically tune read mapping parameters. The motivation is strong: default mapping parameters are often suboptimal for specific downstream tasks or emerging sequencing platforms. The dual-stage simulation approach, which captures sequence-context-dependent biases and K-mer error models, is computationally elegant and demonstrates improved fidelity over existing simulators like NanoSim and BadRead.

However, the manuscript exhibits critical gaps in its evaluation pipeline, rendering the conclusions about "systematic optimization" somewhat premature. Specifically, the framework neglects comprehensive small variant assessment (particularly Indels) and relies on an outdated caller for SNPs. Furthermore, there is a substantial risk of circular reasoning (overfitting to the simulator), and the claims of being a "tool-agnostic" framework lack empirical support. A major revision is required to validate the framework's robustness across modern, state-of-the-art bioinformatics workflows.
Response:
We appreciate the reviewer's positive assessment of the motivation and design of CycSim, and we agree that the original manuscript did not sufficiently address several important aspects of the evaluation. We have therefore substantially revised both the analyses and the presentation.

First, we added Clair3-based SNP and indel calling to complement the original Longshot-based SNP evaluation. For Cyclone data, because no publicly available Cyclone-specific Clair3 model is currently available, we used the ONT-compatible r941_prom_hac_g360+g422 model as a unified alternative. We note that this pretrained model was developed based on ONT-style data and machine-learning mapping conventions; thus, its absolute predictive accuracy is inherently bounded when applied to a different sequencing chemistry like Cyclone. This additional analysis directly addresses the concern that indels were omitted and that Longshot alone is not sufficient for modern long-read small-variant benchmarking (Lines 171-176, Tables S1-S2). Second, we added additional validation datasets and analyses to reduce the risk of simulator-specific overfitting, including held-out HG002 chromosomes, whole-genome HG002 validation, independent HG005 small-variant validation, and a CHM13-based synthetic SV benchmark (Lines 165-183, 206-209, Tables S1-S9). Third, we revised the "tool-agnostic" claim to avoid overstatement, we have added an expanded discussion of the advantages, limitations, and appropriate use cases of the optimization framework (Lines 217-267).

Major comments
1. Inadequate assessment of small variants and outdated downstream tooling
The manuscript emphasizes CycSim's superiority in modeling localized, context-dependent errors in simple STRs. Ironically, these low-complexity regions are exactly where small variants (especially insertions and deletions) suffer the most from long-read misalignment. When evaluating the "general-purpose alignment," the authors only benchmarked SNP and SV accuracy. Indels, which represent the primary error modality in nanopore sequencing and the most challenging variant class for mappers, are entirely omitted from the evaluation.
Response:
We agree with the reviewer. Indel evaluation is essential for assessing long-read mapping performance, particularly because low-complexity and repetitive regions are enriched for insertion/deletion errors and are sensitive to alignment parameter choices. In the revised manuscript, we therefore incorporated indel evaluation into the general-purpose mapping assessment.

Specifically, we added Clair3-based SNP and indel calling using default parameters and benchmarked the resulting calls with hap.py using the vcfeval engine. Because no publicly available Cyclone-specific Clair3 model is currently available, we used the ONT-compatible r941_prom_hac_g360+g422 model for Cyclone data. These analyses were added to the Cyclone general-purpose parameter evaluation (Tables S1-S2), and the main text has been revised to state explicitly that SNP, indel, and SV benchmarks were used to assess general-purpose mapping performance (Lines 165-183).

2. The authors utilized Longshot for SNP calling. Longshot is an older tool inherently limited to diploid SNVs and is incapable of calling Indels. In the current era of long-read sequencing, the standard practice for small variant calling relies heavily on deep learning models (e.g., DeepVariant, Clair3, or longcallD). The authors must incorporate Indel evaluation into their objective function and benchmarking. Especially on BGI Cyclone data and ONT GIAB data(which are release public available online)
Response:
We agree with the reviewer that modern small-variant evaluation should include indels and should not rely solely on Longshot. In the revised manuscript, we therefore added Clair3-based SNP and indel calling to complement the original Longshot-based SNP evaluation.

For Cyclone data, because no publicly available Cyclone-specific Clair3 model is currently available, we used the ONT-compatible r941_prom_hac_g360+g422 model as the closest available alternative. We note that this pretrained model was developed using ONT-style data and mapping conventions, and its predictions may therefore partly reflect map-ont-like alignment characteristics. In this sense, applying this model directly to BAM files generated with our newly optimized Cyclone parameters represents a conservative evaluation. A Clair3 model retrained or fine-tuned using Cyclone data aligned with the optimized parameters may provide a more platform-matched small-variant evaluation, but such caller retraining was beyond the scope of the present study.

These analyses show that the optimized Cyclone parameters maintained comparable Clair3-based SNP and indel performance while substantially reducing mapping runtime (Lines 165-183, Tables S1-S2). We also revised the Results and Methods sections to clarify that general-purpose mapping performance was evaluated using SNP, indel, and SV benchmarks (Lines 171-178, 329-334).

Because no new ONT/HiFi general-purpose parameter set was recommended in this study, we focused the added Clair3-based SNP/indel analysis on Cyclone.

3. The optimization workflow relies on CycSim-simulated data to maximize a composite metric, and then the optimized parameters are screened on empirical data. Because the Bayesian optimizer is trained exclusively on CycSim outputs, the "optimal" parameters might merely be those that overfit CycSim's specific mathematical error models, rather than reflecting universal biological alignment truths.
Response:
This is a good point, and simulator-specific overfitting is a central concern for simulation-guided optimization. We therefore strengthened the validation strategy in the revised manuscript. Parameter

search was performed only on CycSim-simulated HG002 chromosomes 1, 2, 17, and 18. The selected parameters were then evaluated on real HG002 data, held-out HG002 chromosomes excluding chromosomes 1, 2, 17, and 18, whole-genome HG002 data, an independent HG005 dataset for small variants, and a CHM13-based synthetic SV benchmark. Small-variant performance was evaluated using Clair3 for SNP/indel calling and Longshot for SNP calling, whereas SV performance was evaluated using two independent long-read SV callers, Sniffles2 and cuteSV. The consistent performance across chromosomes, samples, benchmark datasets, and variant-calling tools supports that the optimized parameters are not simply overfitted to the simulated training chromosomes, a single sample, or a specific software (Lines 165-183, 197-209, Tables S1-S9).

4. Regarding writing and presentation, the manuscript suffers from a structural disconnect, reading somewhat like two disjointed projects. The authors should explicitly bridge this gap by explaining why high-fidelity simulation is a strict prerequisite for Bayesian optimization (e.g., to prevent the optimizer from overfitting to the artifactual error distributions produced by traditional simulators). Methodologically, crucial details are opaque; the authors must provide the exact mathematical formulation and weightings for the "composite accuracy metric" and explicitly define the Optuna hyperparameter search bounds (e.g., ranges for k, w, A, B, O, E). Furthermore, when reporting relative performance gains in the main text (e.g., "2.78-fold faster"), the exact baseline preset being compared against must be clearly specified to avoid ambiguity. Finally, several typographical and grammatical errors require correction prior to publication, including misspellings ("framwork",
"charaterizes", "accross"), a syntax error due to an incorrect period ("...uniform error distributions. CycSim..."), and a tense inconsistency in the authors' contributions ("review" instead of "reviewed").
Response:
We thank the reviewer for these constructive suggestions.

To bridge the simulation and optimization components, we added an explicit rationale explaining "High-fidelity simulation is a prerequisite for simulation-guided optimization because an optimizer may otherwise overfit to simulator-specific artifacts, such as artificially uniform error distributions or missing sequence-context-dependent biases. Leveraging the improved realism of CycSim-generated reads..." (Lines 137-141).

We also expanded the Methods section to provide the exact formulation of the optimization objective and the full minimap2 search space. "For simulation-based parameter search, each minimap2 configuration was evaluated against the known truth alignment of simulated reads. Only primary alignments were retained. Two metrics were calculated: interval accuracy, defined as the fraction of reads whose predicted reference interval matched the truth interval within 50 bp, and CIGAR-operation accuracy, defined as the fraction of comparable read positions with identical CIGAR-derived operation labels between the predicted and truth alignments. The general-purpose mapping score was calculated as the average of interval accuracy and CIGAR-operation accuracy, and Optuna minimized one minus this score. Mapping runtime was recorded for each configuration and used during empirical screening. The minimap2 search space included -k = 15–19, -w = 10–19, -A = 1–2, -B = 3–9, -O = 4–14,15–49, -E = 2–3,1, -s ∈ {30, 40, 80, 100, 150, 180, 200, 240}, and -U = 10–80,{500, 5000, 50000, 500000, 1000000}. After optimization, the top 40 non-redundant configurations were re-evaluated on 30× real HG002 data from chromosomes 1, 2, 17, and 18 using downstream SNP, Indel, and SV benchmarks. For SV-oriented optimization, the same minimap2 search space was used, but each candidate configuration was evaluated directly using downstream SV-calling performance. For each configuration, SVs were called with Sniffles2 and benchmarked with Truvari, and Optuna minimized one minus the SV F1 score. cuteSV was used for independent caller validation." (Lines 348-370).

We clarified that the 2.78-fold speedup for Cyclone data was measured relative to minimap2's ONT-oriented map-ont preset (Lines 22, 160-161).

Finally, we corrected the typographical and grammatical errors noted by the reviewer, including "framework," "characterizes," "across," the incorrect period before "CycSim," and the authors' contribution tense inconsistency.

Minor

1. For the newly developed Cyclone platform, the authors used the minimap2 map-ont preset as the baseline, reporting a 2.78-fold increase in mapping speed after optimization. Given that Cyclone represents a distinct chemistry and signal profile from Oxford Nanopore, using an ONT-specific preset as the starting baseline might artificially inflate the magnitude of the improvement.
Response:
We agree that this comparison requires careful wording. At the time of this study, no established minimap2 preset specifically designed for Cyclone data was available. We therefore used minimap2's ONT-oriented map-ont preset as an initial baseline, because Cyclone, like ONT, is a nanopore-based long-read sequencing platform, and map-ont represents a reasonable starting point for nanopore-like long reads in the absence of a Cyclone-specific preset.

To avoid overstatement, we revised the manuscript to explicitly state that the 2.78-fold speedup was measured relative to minimap2's ONT-oriented map-ont preset (Lines 22, 160-161).

2. Figure 1 Clarifications: In Figure 1C, the legend mentions an "empirical upper bound" for the Raw data. It would be helpful to briefly explain the statistical rationale for how this splitting approach yields a valid upper bound in the main text or supplement.
Response:
We added an explanation to the main text. "For the Raw comparison, real reads were randomly split into two subsets and compared against each other. Because both subsets originated from the same empirical sequencing dataset, their similarity represents an empirical upper bound for the expected concordance between simulated and real reads under finite sampling" (Lines 102-106).

3. Bayesian optimization involving thousands of minimap2 runs is computationally intensive. Please provide a summary of the computational resources (e.g., CPU hours, peak memory usage) required to run the full four-stage pipeline. This is crucial for readers to assess the practical accessibility of this framework.
Response:
Thank you for the suggestion. We added Table S11 summarizing the computational resources required for the full optimization workflow. We also added a discussion emphasizing that the full optimization process is most suitable for large-scale or repeatedly used workflows, or for software developers aiming to establish optimized default parameters for specific platforms and analysis tasks (Lines 254-258).

Reviewer #2: In this work, Hu et al describe CycSim, a context aware simulator for diverse long read chemistries. Using this simulator, the authors aimed to optimize the mapping parameters to improve mapping speed and accuracy of variant calls.

This is important, given the increase in use of long read sequencing, particularly in the wake of upcoming technologies such as Cyclone. However, I have significant concerns about how the results are analyzed and work is presented. Despite it being a short article, I had to do a lot of back and forth reading due to lack of clarity in presentation. It would also help to have line numbers to point out specific places in the manuscript. Further I have some questions which the authors should address with a revision.
Response:
We thank the reviewer for recognizing the importance of this work. We have substantially reorganized and expanded the manuscript to improve clarity. We added a clearer connection between CycSim's simulation fidelity and the downstream parameter optimization framework, expanded the Methods section, revised

figure presentation, added additional validation analyses, and clarified the limitations of the framework. We will also ensure that the resubmitted manuscript contains line numbers to facilitate review.

CycSim is a "dual-stage framework" - Does that mean the users are expected to train with every new sample? Or the trained model can now simulate reads for any sample? How do we know the training is not over engineered for the HG002 sample, particularly the 4 chromosome subset?
Response:
This is a good question. CycSim does not require retraining for every individual sample if an existing model was trained on data generated from the same sequencing platform and chemistry, and from a closely related genomic context. However, when the sequencing platform, chemistry, library preparation protocol, or genome k-mer profile differs substantially, retraining is recommended to better capture the relevant empirical error characteristics. To improve robustness, CycSim provides a user-defined parameter for introducing random errors according to the global error-rate distribution, which helps maintain global error consistency and increase error-pattern diversity. For k-mer contexts absent from the training data, CycSim automatically falls back to the global average error model rather than terminating the simulation. This preserves the overall error level, although some fine-scale context-specific biases may still be missed (Lines 217-226).

To address the concern that the model might be over-engineered to HG002 chromosomes 1, 2, 17, and 18, we added additional validation analyses. Although the CycSim model was trained using only the selected HG002 chromosomes, its performance was further evaluated on an independent HG005 Cyclone dataset. The simulated HG005 reads showed alignment identity distributions and substitution profiles similar to those of real HG005 reads, supporting that the trained model was not merely overfitted to the HG002 training sample (Lines 120-124, Fig. S8).

We also added a limitations paragraph clarifying that CycSim is expected to perform best when the training data are generated from the same or a closely related genome, sequencing platform, chemistry, and library preparation protocol as the intended simulation target (Lines 130-133). In addition, we clarified that when the target genome contains k-mer contexts absent from the training data, CycSim uses the global average error model as a fallback rather than terminating the simulation. This preserves the overall error rate but may lose some systematic context-specific error biases (Lines 223-226).

The algorithm characterizes many aspects of the read including strand, orientation etc. Are they used for training in any way?
Response:
Yes. These read-level properties are learned during the model-training stage and sampled during read simulation. CycSim learns empirical distributions of read structure, including strand orientation, chimerism, aligned and unaligned lengths, and alignment identity. During simulation, these learned distributions are used to define structural composition, expected error rate, and complete read structure of synthetic reads. The k-mer and error-transition models then determine the local base-level error process in the aligned core.

Are the kmer models built for each "kind" of genomic region (for example LCRs/TRs)?
Response:
CycSim does not build separate explicit models for predefined genomic annotations such as LCRs or TRs. Instead, CycSim learns k-mer-specific error profiles from empirical alignments. Because low-complexity and repeat regions have distinctive sequence-context compositions, their error characteristics are captured through the corresponding k-mer contexts and the error-transition model. This design avoids requiring fixed region annotations while still allowing localized sequence-context effects to be modeled.

Simulation/validation is done for the same set of chromosomes as those that were used for training. How do the various parameters benchmarked in Fig 1 fare for other chromosomes which the model has not seen?
Response:
We agree that performance beyond the training chromosomes is an important issue.

First, we added an independent validation using HG005 Cyclone data. Although the CycSim model was

trained using HG002 chromosomes 1, 2, 17, and 18, applying this HG002-trained model to the independent HG005 dataset generated simulated reads with alignment identity distributions and substitution profiles similar to those of real HG005 reads, supporting that the trained model was not merely overfitted to the HG002 training sample (Lines 120-124, Fig. S8).

Second, we clarified that a model trained on only four chromosomes is not expected to capture all chromosome-specific k-mer contexts across the whole genome. Other chromosomes may contain k-mer contexts, repeat structures, or local sequence compositions that are absent in the training chromosomes. For such unseen k-mer contexts, CycSim does not terminate the simulation; instead, it falls back to the global average error model. This fallback preserves the overall error-rate distribution, but it may miss systematic context-specific error biases in those unseen regions. As a result, the context-aware advantage of CycSim may be reduced in genomic regions whose k-mer composition differs substantially from the training set.

We therefore revised the manuscript to state that CycSim is expected to perform best when the training data are generated from the same or a closely related genome, sequencing platform, chemistry, and library preparation protocol as the intended simulation target. When the target genome or chromosome set differs substantially from the training data, retraining with representative data is recommended to better capture the relevant empirical error characteristics (Lines 130-133, 217-226).

In LCRs, CycSim outperforms other tools. This is a crucial point. But what is shown is mapping identity - as far as I know, the mapping identity in these regions should be lower. Not sure why it's higher, unless I'm misunderstanding how this is calculated. Also, it would be important to show other features of the reads such as kmer profiles and substitutions, specifically in various genomic regions, rather than mapping identity % alone.
Response:
We thank the reviewer for raising this point. We agree that the interpretation of Fig. 1D and Fig. S3 needed to be clarified. In these figures, the lower heatmap represents STR/SSR density, and regions with lower SSR density generally show higher alignment identity. For example, although the chromosome 1 centromeric region is broadly repetitive, its local SSR density, defined by 1-6 bp motifs with at least three repeat units, is lower than that of other regions, which explains the higher mapping identity observed in that region. Therefore, these figures show that CycSim better recapitulates the positional heterogeneity of alignment identity associated with local SSR density.

To provide a more direct genome-wide assessment, we added SSR-stratified error analyses comparing regions with high and low SSR content. We decomposed errors into mismatch, insertion, and deletion components and performed region-stratified substitution/error analyses across sequence-complexity classes. These analyses showed that the increased error rate in SSR-rich regions of real ONT and Cyclone data was mainly driven by elevated deletion rates, and that CycSim reproduced these region-specific error profiles more closely than existing simulators (Figs. S4-S6).

We revised the text accordingly to clarify the interpretation of Fig. 1D and Fig. S3 and to highlight the newly added genome-wide SSR-stratified analyses (Lines 108-116).

Regarding the parameter tuning/optimization - I have significant problems with how the data is presented. First of all, radar charts, while they look fancy, are less effective in depicting small changes, which is what the authors are trying to show here. Simple bar charts would have been way more clear. Also, this feels like an independent goal and section, and the connection with their simulation strategy is not clear.
Response:
We thank the reviewer for this helpful suggestion. We replaced the original radar charts with clearer bar-style visualizations and added numerical labels where appropriate (Figs. 2F, 2H, and S10). We also included the underlying plotting data in supplementary tables to make the quantitative comparisons more transparent and easier to verify (Tables S4-S9).

In addition, we revised the manuscript to explicitly connect the parameter-optimization section with the simulation strategy, “High-fidelity simulation is a prerequisite for simulation-guided optimization because an optimizer may otherwise overfit to simulator-specific artifacts, such as artificially uniform error

distributions or missing sequence-context-dependent biases. Leveraging the improved realism of CycSim-generated reads...” (Lines 137–141).

More importantly, why wasn't this done with reads simulated with other tools? For all we know, the optimization may have worked with reads simulated by BadRead or PBSim as well.
Response:
This is an important question. In principle, the Bayesian optimization framework can use reads generated by any simulator. However, the reliability of simulation-guided optimization depends directly on how well the simulated reads reproduce real read characteristics. Our benchmarking showed that existing simulators differ from real data in several important aspects, including global error-rate distributions, k-mer error profiles, and localized error heterogeneity associated with low-complexity regions (Figs. 1, S2-S6). Therefore, optimizing parameters using lower-fidelity simulations could select configurations that perform well on simulator-specific artifacts but do not necessarily generalize to real data.

We have therefore clarified in the revised manuscript, “the optimization framework is modular and can in principle incorporate reads generated by other simulators. Because simulation-guided optimization depends on how well simulated reads reflect real read characteristics, parameters selected from any simulator should still undergo empirical screening, independent validation, and, where necessary, additional diagnostic evaluation to avoid simulator-specific biases” (Lines 258-264).

The 2.78-fold increase in speed for Cyclone data is compared to map-ont preset, which by definition is not optimized for Cyclone data. While this is an important exercise and result, not sure how this is a direct benefit of CycSim. Could the mapping speed be not optimized by trying Optuna on raw Cyclone data directly?
Response:
At the time of this study, no established minimap2 preset specifically designed for Cyclone data was available. We therefore used minimap2’s ONT-oriented map-ont preset as an initial baseline, because Cyclone, like ONT, is a nanopore-based long-read sequencing platform, and map-ont represents a reasonable starting point for nanopore-like long reads in the absence of a Cyclone-specific preset. To avoid overstatement, we revised the manuscript to explicitly state that the 2.78-fold speedup was measured relative to minimap2’s ONT-oriented map-ont preset (Lines 22, 160-161).

Regarding direct Optuna optimization on raw Cyclone data, raw reads lack known true alignment coordinates and base-level truth CIGAR operations. Therefore, direct optimization on raw data alone cannot compute the interval-level and CIGAR-operation accuracy metrics used in the simulation-based stage. This distinction is important because, for general-purpose mapping optimization, SNP and SV calling were used only as downstream validation to confirm that the newly selected mapping parameters did not compromise variant-calling performance. They were not the direct optimization targets. Instead, the optimization objective was to improve alignment accuracy and runtime, which requires known ground-truth read coordinates and alignment operations.

One could optimize directly against downstream variant-calling metrics on real data, but this would represent a task-specific optimization rather than general-purpose mapping optimization. It would also be much more computationally expensive for thousands of parameter trials and could overfit to the specific benchmark regions, truth set, or variant caller used during optimization. Our framework therefore uses CycSim-generated reads to efficiently generate candidate parameter sets with known ground truth, followed by empirical screening and independent validation on real data.

The authors claim that the framework is robust in optimizing parameters "across diverse sequencing platforms". But in their own words, the gains for PacBio and ONT were <0.1%.
Response:
We agree with the reviewer that the original wording could be interpreted as overstating the gains for general-purpose mapping across mature sequencing platforms. For ONT and HiFi data, the existing minimap2 presets have already been extensively optimized by the minimap2 developers and represent strong general-purpose defaults. Therefore, it is not unexpected that our framework identified only marginal accuracy gains (<0.1% in simulation) with slightly reduced runtime for these mature platforms.

Importantly, this result also supports the validity of the optimization framework: when existing presets are already well matched to the data, the framework does not force substantially different parameter settings, but instead identifies configurations with performance close to the developer-optimized defaults.

We have therefore revised the manuscript to clarify this point, “Conversely, for HiFi and ONT datasets, the framework identified configurations with performance close to the established minimap2 default presets. Because these presets have already been extensively optimized for mature long-read platforms, only marginal accuracy gains (<0.1% in simulation) with slightly reduced runtime were observed (Fig. S9). These results indicate that the framework can recover parameter settings comparable to developer-optimized defaults when existing presets are already well matched to the data, while still enabling parameter refinement for datasets with distinct read characteristics.” (Lines 178-186).

The truvari refine parameters had a flag -p 0.0, indicating only position (that too up to 1kb distance) was taken into account when calculating the overlap, irrespective of sequence similarity. Most people in practice keep 0.5-0.7, often with reciprocal overlap. Curious to see how the results change with such parameters.
Response:
We thank the reviewer for pointing this out. We re-evaluated the SV benchmarking workflow and removed the permissive -p 0.00 setting. In the revised manuscript, SVs were benchmarked using Truvari with --passonly -r 1000 --refine, and with default sequence-similarity threshold (-p 0.7). The revised results show the same overall trends: SV-oriented mapping parameters reduce runtime and preserve or improve SV F1 scores across platforms, coverage depths, and both Sniffles2 and cuteSV (Lines 328-329, Tables S3-S9).

It is not clear whether the SNP and SV optimized parameters are the same or not. If yes, this should be clarified. If not, authors should include results on what happens to SNP accuracy when using SV specific parameters. While I agree that there is merit in using specific alignment parameters for specific tasks, in practice, users might just use the same BAM file for multiple types of variants - hence having this information can help the user in deciding which parameters they want to use.
Response:
This is an exceptionally constructive suggestion. The general-purpose optimized parameters and the SV-oriented optimized parameters are indeed not identical. The former maximizes overall alignment accuracy and runtime, whereas the latter selectively optimizes macro-interval consistency and breakpoint placement to favor SV detection.

To fully address the practical scenario where users may apply a single BAM file to multiple downstream variant types, we executed a cross-task evaluation by performing Clair3-based SNP/Indel calling on alignments generated by our SV-oriented optimized parameters (Table S10). As the reviewer astutely anticipated, while the SV-oriented parameters improved SV-calling efficiency and runtime, they led to a minor fluctuation in single-base small variant accuracies in certain depth combinations (e.g., a subtle decrease in ONT 15x Indel F1 score from 0.5932 to 0.5905). This minor drop reflects the inherent heuristic trade-offs in long-read mappers, where loosening seed or gap penalties to aggregate fragmented alignments across large structural disruptions can occasionally distort local, single-base resolution.
To leverage this finding into a constructive workflow for users, we have fundamentally restructured the Discussion section (Lines 228-240) by proposing a comprehensive "Best Practice Guideline". We explicitly state that for projects focused primarily on structural variations and cohort screening, the SV-optimized parameters offer maximum efficiency; whereas for holistic pan-variant exploration requiring high-precision single-nucleotide resolution, the CycSim-derived general-purpose preset remains the optimal choice.

The gains in F1 scores are marginal. Authors should discuss if and why such marginal improvements are important.
Response:
We agree with the reviewer that the SV F1-score improvements are modest in absolute value and should be discussed more carefully. Although the increases in SV F1 score were relatively small, the optimized parameters reduced mapping runtime by 8.14-34.16% across ONT, HiFi, and Cyclone datasets. Such speed improvements can be important for large-cohort or population-scale long-read projects. In this setting, even modest F1-score gains are useful because they are achieved together with substantial computational savings rather than at the cost of accuracy.

We also evaluated the effect of SV-oriented parameters on small-variant calling. Clair3-based SNP and indel results showed that small-variant performance was only slightly affected in most settings, although SV-oriented parameters did not consistently improve SNP and indel calling across all platform-depth combinations (Table S10). In addition, low-depth long-read datasets are generally less suitable for reliable SNP/indel calling, and in such cases SV detection and mapping efficiency may be the primary analysis goals.

We therefore revised the Discussion to describe the appropriate use cases for SV-oriented parameters more explicitly (Lines 233-240).

Other comments:

What happens when you simulate high coverage? Would it recapitulate actual high coverage data or would there be repeated data due to limits of a kmer model?
Response:
We agree with the reviewer that when the simulation coverage drastically exceeds the training depth, the localized K-mer specific errors inherent to the training data could become over-represented. CycSim samples error profiles from empirical training data, and k-mer-specific error models are estimated from a finite training set. Therefore, when the simulated depth substantially exceeds the training depth, the simulator will continue sampling genomic positions and reads, but some learned k-mer-specific error contexts may be over-represented. This does not mean that identical reads are simply duplicated; rather, the diversity of context-specific error patterns may become limited by the finite training data.

To mitigate this effect, CycSim provides a user-defined parameter for introducing random errors according to the global error-rate distribution, which helps maintain global error consistency and increase error-pattern diversity at high simulated coverage. We added this limitation and mitigation strategy to the revised manuscript (Lines 218-226).

Was the simulation done multiple times? This should be clarified. If not done, it should be - to see how reproducible the results are.
Response:
We performed three independent CycSim simulations using the same trained model and simulation settings. The three replicates produced nearly overlapping mapping identity distributions and highly consistent substitution spectra, supporting the reproducibility of CycSim-generated read profiles (Fig. S7, Lines 116-120).

Thanks for providing the optimized parameters for each platform - can there be a comment on why the authors think these parameters outperform default parameters?
Response:
We thank the reviewer for this suggestion. For SV-oriented optimization, the final parameter sets were selected because they achieved improved SV F1 scores together with reduced mapping time. For Cyclone general-purpose mapping, the optimized parameter set was selected because it substantially reduced mapping time while maintaining comparable alignment and variant-calling performance.

We clarified this selection criterion and provided the final optimized minimap2 parameter sets in the revised Methods: “The optimal minimap2 parameters identified for SV calling were selected based on improved F1 score and reduced mapping time. For ONT data, the optimized parameters were -k 21 -w 21 -A 1 -B 9 -O 13,44 -E 3,1 -s 30 -U 20,50000. For HiFi data, the optimized SV-calling parameters were -k 23 -w 22 -A 1 -B 9 -O 13,41 -E 3,1 -s 180 -U 10,5000. For Cyclone data, the optimal parameters for general alignment were -k 16 -w 13 -A 2 -B 4 -O 4,41 -E 2,1 -s 180 -U 70,1000000, which substantially reduced mapping time while maintaining comparable alignment accuracy. In contrast, the Cyclone parameters optimized specifically for SV calling were -k 17 -w 13 -A 1 -B 9 -O 13,44 -E 3,1 -s 30 -U 70,500, which achieved a higher SV F1 score together with faster mapping.” (Lines 372-382).

Minor:

Several typos - such as "framwork", "charaterize", and missing commas etc.

Response:
We corrected the typographical and grammatical errors noted by the reviewer, including “framework,” “characterizes,” “across,” the incorrect period before “CycSim,” and the authors’ contribution tense inconsistency.

I could not initially find cutesv results, till I stumbled upon them in the tables. Please tag the table numbers at the appropriate place in the text.
Response:
Thank you for the suggestion. We revised the Results section to explicitly state that the optimization trends were consistent across both Sniffles2 and cuteSV, and we added the corresponding table citations at the relevant locations in the text (Tables S4-S9).

Editor Comments:

GigaScience has also published a number of relevant papers on metadata standardization, FAIR data practices, and reproducible workflows in biotechnology that may help contextualize your work. We have listed several recent examples below for your reference. Citing some of these articles—where appropriate in the Background or Discussion—would help frame your contribution within ongoing community efforts and strengthen the presentation of related tools and frameworks.
1. Gaitán N, Duitama J. A graph clustering algorithm for detection and genotyping of structural variants from long reads[J]. GigaScience, 2024, 13: giad112. https://doi.org/10.1093/gigascience/giad112

--
Please also take a moment to check our website at https://www.editorialmanager.com/giga/l.asp?i=243456&l=T001G7WV for any additional comments that were saved as attachments.

Response: We sincerely thank the Editor for recommending this highly relevant and excellent work on structural variant detection from long reads (Gaitán and Duitama, 2024). We have thoroughly read the paper and cited it in the Introduction (Line 42) of our revised manuscript to better contextualize our SV-oriented parameter optimization framework within ongoing community efforts.

em Editorial Manager®

Cookie Preference Center

We use cookies which are necessary to make our site work. We may also use additional cookies to analyze, improve and personalize our content and your digital experience. For more information, see our Cookie Policy.

You may choose not to allow some types of cookies. However, blocking some types may impact your experience of our site and the services we are able to offer. See the different category headings below to find out more or change your settings.

Allow all

Manage Consent Preferences

Strictly Necessary Cookies

Always active

These cookies are necessary for the website to function and cannot be switched off in our systems. They are usually only set in response to actions made by you which amount to a request for services, such as setting your privacy preferences, logging in or filling in forms. You can set your browser to block or alert you about these cookies, but some parts of the site will not then work.

Functional Cookies

These cookies enable the website to provide enhanced functionality and personalisation. They may be set by us or by third party providers whose services we have added to our pages. If you do not allow these cookies then some or all of these services may not function properly.

Performance Cookies

These cookies allow us to count visits and traffic sources so we can measure and improve the performance of our site. They help us to know which pages are the most and least popular and see how visitors move around the site.

Targeting Cookies

These cookies may be set through our site by our advertising partners. They may be used by those companies to build a profile of your interests and show you relevant adverts on other sites. If you do not allow these cookies, you will experience less targeted advertising.

Cookie List

Clear

Apply Cancel

Consent Leg.Interest

Confirm my choices

Powe
