# Supplementary material for: Context-aware simulation enables systematic optimization of long-read mapping parameters
Source: Gigascience. 2026 Jul 8;15:giag079. doi: 10.1093/gigascience/giag079 (PMC13401045; doi:10.1093/gigascience/giag079)
Supplement: giag079_Authors_Response_To_Reviewer_Comments_revision_1 [file giag079_authors_response_to_reviewer_comments_revision_1.pdf]

Author's Response To Reviewer Comments

Dear Editor and Reviewer,
We sincerely thank the Editor and Reviewer for the positive assessment of our revised manuscript and for the remaining constructive suggestion. We have carefully addressed the remaining point as described below.

Editor comment: Please register any new software application in the bio.tools and SciCrunch.org databases to receive RRID and biotoolsID identifiers, and include these in your manuscript.

Response:
As described in the previous revision, CycSim has been registered in both bio.tools and SciCrunch. It has been assigned the biotoolsID cycsim and RRID SCR_028425. These identifiers have been included in the "Availability of Source Code and Requirements" section of the revised manuscript.

Reviewer #1

Comment: The authors have addressed most of my concerns by adding Clair3-based SNP and indel evaluation. My only remaining concern is that the Clair3 model used for Cyclone data is ONT-trained, which may not fully match Cyclone error profiles. If feasible, I suggest adding a platform-matched evaluation, such as longcallD or a Clair3 model retrained/fine-tuned on Cyclone data.

Response:
We thank the reviewer for this thoughtful suggestion. We agree that a platform-matched small-variant caller would be ideal for Cyclone data. However, to our knowledge, no publicly available Cyclone-specific Clair3 model is currently available. We also contacted the Cyclone team regarding this issue, and they confirmed that no public Cyclone-specific Clair3 model is available at present and suggested using the ONT-compatible Clair3 model as the closest available alternative. Therefore, we used the ONT-compatible r941_prom_hac_g360+g422 Clair3 model for Cyclone SNP and indel calling.

Although this model is not Cyclone-specific, our results suggest that it provides a reasonable small-variant evaluation for the current study. First, the Clair3-based SNP and indel results on Cyclone data showed comparable high accuracy for both the optimized and baseline mapping parameters (Table S1). Second, the observed Cyclone error profiles were similar to ONT error profiles in several analyses, including substitution bias patterns and region-stratified error decomposition (Fig. 1B, Fig. S4, and Fig. S6). These results suggest that the ONT-compatible Clair3 model is unlikely to substantially bias the relative comparison between Cyclone mapping parameter sets in this study.

Nevertheless, we agree that a Cyclone-specific caller model would provide a more platform-matched evaluation. Retraining or fine-tuning Clair3 on Cyclone data, or evaluating with a Cyclone-validated longcallD model, would require a sufficiently curated platform-specific training set and was beyond the scope of the present revision.

Editorial Manager®

Cookie Preference Center

We use cookies which are necessary to make our site work. We may also use additional cookies to analyze, improve and personalize our content and your digital experience. For

more information, see our Cookie Policy.

You may choose not to allow some types of cookies. However, blocking some types may impact your experience of our site and the services we are able to offer. See the different category headings below to find out more or change your settings.

Allow all

Manage Consent Preferences

Strictly Necessary Cookies

Always active

These cookies are necessary for the website to function and cannot be switched off in our systems. They are usually only set in response to actions made by you which amount to a request for services, such as setting your privacy preferences, logging in or filling in forms. You can set your browser to block or alert you about these cookies, but some parts of the site will not then work.

Functional Cookies

These cookies enable the website to provide enhanced functionality and personalisation. They may be set by us or by third party providers whose services we have added to our pages. If you do not allow these cookies then some or all of these services may not function properly.

Performance Cookies

These cookies allow us to count visits and traffic sources so we can measure and improve the performance of our site. They help us to know which pages are the most and least popular and see how visitors move around the site.

Targeting Cookies

These cookies may be set through our site by our advertising partners. They may be used by those companies to build a profile of your interests and show you relevant adverts on other sites. If you do not allow these cookies, you will experience less targeted advertising.

Cookie List

Clear

Apply Cancel

Consent Leg.Interest

Confirm my choices

Powe[illegible]
