# Supplementary material for: Context-aware simulation enables systematic optimization of long-read mapping parameters
Source: Gigascience. 2026 Jul 8;15:giag079. doi: 10.1093/gigascience/giag079 (PMC13401045; doi:10.1093/gigascience/giag079)
Supplement: giag079_Reviewer_1_Report_Original_Submission — Reviewer 1 -- 4/2/2026 [file giag079_reviewer_1_report_original_submission.pdf]

Reviewer Report

Title: Context-aware simulation enables systematic optimization of long-read mapping parameters

Version: Original Submission **Date:** 4/2/2026

Reviewer name: Reviewer 1

Reviewer Comments to Author:

The authors present CycSim, a context-aware long-read simulator, and a Bayesian optimization framework designed to systematically tune read mapping parameters. The motivation is strong: default mapping parameters are often suboptimal for specific downstream tasks or emerging sequencing platforms. The dual-stage simulation approach, which captures sequence-context-dependent biases and K-mer error models , is computationally elegant and demonstrates improved fidelity over existing simulators like NanoSim and BadRead.

However, the manuscript exhibits critical gaps in its evaluation pipeline, rendering the conclusions about "systematic optimization" somewhat premature. Specifically, the framework neglects comprehensive small variant assessment (particularly Indels) and relies on an outdated caller for SNPs. Furthermore, there is a substantial risk of circular reasoning (overfitting to the simulator), and the claims of being a "tool-agnostic" framework lack empirical support. A major revision is required to validate the framework's robustness across modern, state-of-the-art bioinformatics workflows.

Major comments

1. Inadequate assessment of small variants and outdated downstream tooling

The manuscript emphasizes CycSim's superiority in modeling localized, context-dependent errors in simple STRs. Ironically, these low-complexity regions are exactly where small variants (especially insertions and deletions) suffer the most from long-read misalignment. When evaluating the "general-purpose alignment," the authors only benchmarked SNP and SV accuracy. Indels, which represent the primary error modality in nanopore sequencing and the most challenging variant class for mappers, are entirely omitted from the evaluation.

2. The authors utilized Longshot for SNP calling. Longshot is an older tool inherently limited to diploid SNVs and is incapable of calling Indels. In the current era of long-read sequencing, the standard practice for small variant calling relies heavily on deep learning models (e.g., DeepVariant, Clair3, or longcallD). The authors must incorporate Indel evaluation into their objective function and benchmarking. Especially on BGI Cyclone data and ONT GIAB data(which are release public available online)

3. The optimization workflow relies on CycSim-simulated data to maximize a composite metric, and then the optimized parameters are screened on empirical data. Because the Bayesian optimizer is trained exclusively on CycSim outputs, the "optimal" parameters might merely be those that overfit CycSim's specific mathematical error models, rather than reflecting universal biological alignment truths.

4. Regarding writing and presentation, the manuscript suffers from a structural disconnect, reading somewhat like two disjointed projects. The authors should explicitly bridge this gap by explaining why high-fidelity simulation is a strict prerequisite for Bayesian optimization (e.g., to prevent the optimizer from overfitting to the artifactual error distributions produced by traditional simulators).

Methodologically, crucial details are opaque; the authors must provide the exact mathematical formulation and weightings for the "composite accuracy metric" and explicitly define the Optuna hyperparameter search bounds (e.g., ranges for k, w, A, B, O, E). Furthermore, when reporting relative performance gains in the main text (e.g., "2.78-fold faster"), the exact baseline preset being compared against must be clearly specified to avoid ambiguity. Finally, several typographical and grammatical errors require correction prior to publication, including misspellings ("framwork", "charaterizes", "accross"), a syntax error due to an incorrect period ("...uniform error distributions. CycSim..."), and a tense inconsistency in the authors' contributions ("review" instead of "reviewed").

Minor

1. For the newly developed Cyclone platform, the authors used the minimap2 map-ont preset as the baseline, reporting a 2.78-fold increase in mapping speed after optimization. Given that Cyclone represents a distinct chemistry and signal profile from Oxford Nanopore, using an ONT-specific preset as the starting baseline might artificially inflate the magnitude of the improvement.
2. Figure 1 Clarifications: In Figure 1C, the legend mentions an "empirical upper bound" for the Raw data. It would be helpful to briefly explain the statistical rationale for how this splitting approach yields a valid upper bound in the main text or supplement.
3. Bayesian optimization involving thousands of minimap2 runs is computationally intensive. Please provide a summary of the computational resources (e.g., CPU hours, peak memory usage) required to run the full four-stage pipeline. This is crucial for readers to assess the practical accessibility of this framework.

Methods

Are the methods appropriate to the aims of the study, are they well described, and are necessary controls included? Choose an item.

Conclusions

Are the conclusions adequately supported by the data shown? Choose an item.

Reporting Standards

Does the manuscript adhere to the journal's guidelines on minimum standards of reporting? Choose an item.

Choose an item.

Statistics

Are you able to assess all statistics in the manuscript, including the appropriateness of statistical tests used? Choose an item.

Quality of Written English

Please indicate the quality of language in the manuscript: Choose an item.

Declaration of Competing Interests

Please complete a declaration of competing interests, considering the following questions:

- Have you in the past five years received reimbursements, fees, funding, or salary from an organisation that may in any way gain or lose financially from the publication of this manuscript, either now or in the future?
- Do you hold any stocks or shares in an organisation that may in any way gain or lose financially from the publication of this manuscript, either now or in the future?
- Do you hold or are you currently applying for any patents relating to the content of the manuscript?
- Have you received reimbursements, fees, funding, or salary from an organization that holds or has applied for patents relating to the content of the manuscript?
- Do you have any other financial competing interests?
- Do you have any non-financial competing interests in relation to this paper?

If you can answer no to all of the above, write 'I declare that I have no competing interests' below. If your reply is yes to any, please give details below.

I declare that I have no competing interests

I agree to the open peer review policy of the journal. I understand that my name will be included on my report to the authors and, if the manuscript is accepted for publication, my named report including any attachments I upload will be posted on the website along with the authors' responses. I agree for my report to be made available under an Open Access Creative Commons CC-BY license (http://creativecommons.org/licenses/by/4.0/). I understand that any comments which I do not wish to be included in my named report can be included as confidential comments to the editors, which will not be published.

I agree to the open peer review policy of the journal

To further support our reviewers, we have joined with Publons, where you can gain additional credit to further highlight your hard work (see: https://publons.com/journal/530/gigascience). On publication of this paper, your review will be automatically added to Publons, you can then choose whether or not to claim your Publons credit. I understand this statement.

Yes Choose an item.
