# Supplementary material for: Context-aware simulation enables systematic optimization of long-read mapping parameters
Source: Gigascience. 2026 Jul 8;15:giag079. doi: 10.1093/gigascience/giag079 (PMC13401045; doi:10.1093/gigascience/giag079)
Supplement: giag079_Reviewer_2_Report_Original_Submission — Reviewer 2 -- 4/15/2026 [file giag079_reviewer_2_report_original_submission.pdf]

Reviewer Report

Title: Context-aware simulation enables systematic optimization of long-read mapping parameters

Version: Original Submission **Date:** 4/15/2026

Reviewer name: Reviewer 2

Reviewer Comments to Author:

In this work, Hu et al describe CycSim, a context aware simulator for diverse long read chemistries. Using this simulator, the authors aimed to optimize the mapping parameters to improve mapping speed and accuracy of variant calls.

This is important, given the increase in use of long read sequencing, particularly in the wake of upcoming technologies such as Cyclone. However, I have significant concerns about how the results are analyzed and work is presented. Despite it being a short article, I had to do a lot of back and forth reading due to lack of clarity in presentation. It would also help to have line numbers to point out specific places in the manuscript. Further I have some questions which the authors should address with a revision.

CycSim is a "dual-stage framework" - Does that mean the users are expected to train with every new sample? Or the trained model can now simulate reads for any sample? How do we know the training is not over engineered for the HG002 sample, particularly the 4 chromosome subset?

The algorithm characterizes many aspects of the read including strand, orientation etc. Are they used for training in any way?

Are the kmer models built for each "kind" of genomic region (for example LCRs/TRs)?

Simulation/validation is done for the same set of chromosomes as those that were used for training. How do the various parameters benchmarked in Fig 1 fare for other chromosomes which the model has not seen?

In LCRs, CycSim outperforms other tools. This is a crucial point. But what is shown is mapping identity - as far as I know, the mapping identity in these regions should be lower. Not sure why it's higher, unless I'm misunderstanding how this is calculated. Also, it would be important to show other features of the reads such as kmer profiles and substitutions, specifically in various genomic regions, rather than mapping identity % alone.

Regarding the parameter tuning/optimization - I have significant problems with how the data is presented. First of all, radar charts, while they look fancy, are less effective in depicting small changes, which is what the authors are trying to show here. Simple bar charts would have been way more clear. Also, this feels like an independent goal and section, and the connection with their simulation strategy is not clear.

More importantly, why wasn't this done with reads simulated with other tools? For all we know, the optimization may have worked with reads simulated by BadRead or PBSim as well.

The 2.78-fold increase in speed for Cyclone data is compared to map-ont preset, which by definition is not optimized for Cyclone data. While this is an important exercise and result, not sure how this is a direct benefit of CycSim. Could the mapping speed be not optimized by trying Optuna on raw Cyclone

data directly?
The authors claim that the framework is robust in optimizing parameters "across diverse sequencing platforms". But in their own words, the gains for PacBio and ONT were <0.1%.
The truvari refine parameters had a flag -p 0.0, indicating only position (that too up to 1kb distance) was taken into account when calculating the overlap, irrespective of sequence similarity. Most people in practice keep 0.5-0.7, often with reciprocal overlap. Curious to see how the results change with such parameters.
It is not clear whether the SNP and SV optimized parameters are the same or not. If yes, this should be clarified. If not, authors should include results on what happens to SNP accuracy when using SV specific parameters. While I agree that there is merit in using specific alignment parameters for specific tasks, in practice, users might just use the same BAM file for multiple types of variants - hence having this information can help the user in deciding which parameters they want to use.
The gains in F1 scores are marginal. Authors should discuss if and why such marginal improvements are important.
Other comments:
What happens when you simulate high coverage? Would it recapitulate actual high coverage data or would there be repeated data due to limits of a kmer model?
Was the simulation done multiple times? This should be clarified. If not done, it should be - to see how reproducible the results are.
Thanks for providing the optimized parameters for each platform - can there be a comment on why the authors think these parameters outperform default parameters?
Minor:
Several typos - such as "framwork", "charaterize", and missing commas etc.
I could not initially find cutesv results, till I stumbled upon them in the tables. Please tag the table numbers at the appropriate place in the text.

Methods

Are the methods appropriate to the aims of the study, are they well described, and are necessary controls included? Choose an item.

Conclusions

Are the conclusions adequately supported by the data shown? Choose an item.

Reporting Standards

Does the manuscript adhere to the journal's guidelines on minimum standards of reporting? Choose an item.

Choose an item.

Statistics

Are you able to assess all statistics in the manuscript, including the appropriateness of statistical tests used? Choose an item.

Quality of Written English

Please indicate the quality of language in the manuscript: Choose an item.

Declaration of Competing Interests

Please complete a declaration of competing interests, considering the following questions:

- Have you in the past five years received reimbursements, fees, funding, or salary from an organisation that may in any way gain or lose financially from the publication of this manuscript, either now or in the future?
- Do you hold any stocks or shares in an organisation that may in any way gain or lose financially from the publication of this manuscript, either now or in the future?
- Do you hold or are you currently applying for any patents relating to the content of the manuscript?
- Have you received reimbursements, fees, funding, or salary from an organization that holds or has applied for patents relating to the content of the manuscript?
- Do you have any other financial competing interests?
- Do you have any non-financial competing interests in relation to this paper?

If you can answer no to all of the above, write 'I declare that I have no competing interests' below. If your reply is yes to any, please give details below.

I declare that I have no competing interests

I agree to the open peer review policy of the journal. I understand that my name will be included on my report to the authors and, if the manuscript is accepted for publication, my named report including any attachments I upload will be posted on the website along with the authors' responses. I agree for my report to be made available under an Open Access Creative Commons CC-BY license (http://creativecommons.org/licenses/by/4.0/). I understand that any comments which I do not wish to be included in my named report can be included as confidential comments to the editors, which will not be published.

I agree to the open peer review policy of the journal

To further support our reviewers, we have joined with Publons, where you can gain additional credit to further highlight your hard work (see: https://publons.com/journal/530/gigascience). On publication of this paper, your review will be automatically added to Publons, you can then choose whether or not to claim your Publons credit. I understand this statement.

Yes Choose an item.
